# From homeostasis to pathology, organelle-specific autophagy in skeletal muscle: a PRISMA-ScR scoping review

**DOI:** 10.3389/fphys.2026.1822139

**Published:** 2026-05-07

**Authors:** Chunxiao Wang, Guanghua Liu, Changsheng Guo, Qi Li, Feixiao Wang, Ming Zhang

**Affiliations:** 1 School of Rehabilitation Medicine, Henan University of Chinese Medicine, Zhengzhou, China; 2 Department of Acupuncture and Moxibusition, Nanjing Hospital of Chinese Medicine Affiliated to Nanjing University of Chinese Medicine, Nanjing, China; 3 School of Rehabilitation Medicine, Heilongjiang University of Chinese Medicine, Heilongjiang, China; 4 4School of Orthopedics and Traumatology, Henan University of Chinese Medicine, Zhengzhou, China; 5 Rehabilitation Diagnosis and Treatment Center, The First Affiliated Hospital of Henan University of Chinese Medicine, Zhengzhou, China

**Keywords:** ER-phagy, lysophagy, mitophagy, nucleophagy, pexophagy, ribophagy, skeletal muscle

## Abstract

**Background:**

As the largest metabolic organ in the human body, skeletal muscle relies on the structural and functional integrity of its organelles for cellular viability and responsiveness. Organelle-specific autophagy, a major subtype of autophagy encompassing mitophagy, pexophagy, reticulophagy (ER-phagy), ribophagy, lysophagy, and nucleophagy, has been reported to exert a protective role in skeletal muscle by selectively eliminating damaged organelles and maintaining cellular homeostasis.

**Objective:**

This scoping review aims to systematically map the current literature on organelle-specific autophagy in skeletal muscle, clarifying the molecular mechanisms, physiological and pathological roles, and research gaps for the six types of organelle-specific autophagy.

**Methods:**

Following the PRISMA-ScR guidelines and the Joanna Briggs Institute framework, we searched PubMed, Embase, Web of Science, and Cochrane Library up to 21 March 2026 using keywords for skeletal muscle combined with mitophagy, pexophagy, ER-phagy, ribophagy, lysophagy, and nucleophagy. Studies involving humans, mice, rats, or skeletal muscle cells were included.

**Results:**

Among 113 included studies, human studies accounted for 15%, animal models 56%, and skeletal muscle cell lines 29%. By autophagy type, mitophagy dominated (87%, 98 studies), reticulophagy and lysophagy each accounted for 4% (five studies each), and lysophagy, pexophagy, ribophagy, and nucleophagy together comprised less than 5%. Regarding evidence level, among 24 human studies, 18 (75%) were cross-sectional observational studies or small case series (level 4), only three were randomized controlled trials (RCTs) (level 2b), and one was an individual RCT (level 1b); the overall evidence was predominantly low-level observational, with a lack of high-quality interventional clinical trials. For autophagic flux methodology, 53% of studies performed dual detection of LC3B and p62, 17% used lysosomal inhibitor blocking experiments, 64% used transmission electron microscopy (TEM) or tandem fluorescent probes, 23% combined bidirectional verification of autophagic function, and 18% examined intervention reversibility. Among 88 animal studies, low risk of bias (RoB) was found in 14 (16%), moderate RoB in 43 (49%), and high RoB in 30 (35%). For 46 cell experiments assessed by five self-established criteria, 83% used TEM to confirm autophagosomes, 28% used lysosomal inhibitors to validate flux, 72% used gene knockout/knockdown to verify mechanisms, 91% used skeletal muscle-derived cell lines, and 41% performed multi-time-point dynamic autophagy detection.

**Conclusions:**

Current research is severely lacking in nonmitophagy mechanisms, standardized dynamic flux assays, and high-quality clinical studies. Furthermore, systematic investigations of sex differences and muscle fiber type specificity are persistently absent, constraining the development of precise intervention strategies. Future efforts should strengthen multiorganelle autophagy network research and clinical translation to provide new targets for preventing and treating skeletal muscle disorders.

## 1 Introduction

Skeletal muscle accounts for approximately 40% of total body weight and is essential for motor control, postural support, thermogenesis, and metabolic balance (Rolfe and Brown, 1997; Baskin et al., 2015). However, when an individual experiences various adverse pathological and physiological stresses, the delicate balance between protein synthesis and degradation in skeletal muscle is disrupted, leading to reduced muscle fiber cross-sectional area, decreased muscle mass, and strength—a process commonly termed skeletal muscle atrophy (Baehr et al., 2022). Skeletal muscle atrophy is not only prevalent in the elderly but can also occur secondary to peripheral nerve injury, cancer, diabetes, heart failure, and other diseases. Thus, based on underlying precipitating factors, skeletal muscle atrophy can be classified as primary or secondary. The addition of a specific code for sarcopenia (M62.84) to the International Classification of Diseases, Tenth Revision, Clinical Modification (ICD-10-CM) in 2016 marked its official recognition as an independent disease (Anker et al., 2016). Sarcopenia not only severely affects patients’ quality of life but is also closely associated with increased morbidity and mortality (Argilés et al., 2016; Landi et al., 2020).

As a terminally differentiated tissue with high metabolism, high protein synthesis, and high mechanical stress, skeletal muscles’ cellular viability and responsiveness are highly dependent on the structural and functional integrity of their organelles, thus demanding stringent organelle quality control (Neel et al., 2013; Tajika et al., 2015). Organelle-specific autophagy, a major form of autophagy, specifically degrades dysfunctional or superfluous organelles to cope with various stresses such as energy deprivation, oxidative stress, and hypoxia, and is a crucial mechanism for maintaining cellular homeostasis (Jin et al., 2013). To date, multiple types of organelle-specific autophagy have been reported, including mitophagy, pexophagy, reticulophagy, ribophagy, lysophagy, and nucleophagy (Yao et al., 2021). Unlike general autophagy, the selective degradation process of organelle-specific autophagy requires specific autophagy receptors to ensure recognition of particular cargoes and abnormal organelles (Kurth et al., 2009).

Therefore, dysfunction in multiorganelle-specific autophagy may constitute a key mechanism in the pathogenesis of skeletal muscle cell injury and functional deterioration, and multiorganelle quality control holds promise as a potential therapeutic target to preserve skeletal muscle viability and improve outcomes of muscle diseases. In this review, we will focus on the detailed molecular mechanisms of organelle-specific autophagy and their synergistic roles in skeletal muscle physiology and pathology.

## 2 Part I: molecular mechanisms of organelle-specific autophagy

Metabolically active skeletal muscle is highly sensitive to organelle damage (Neel et al., 2013; Tajika et al., 2015). Unlike general autophagy, organelle-specific autophagy precisely eliminates damaged mitochondria, endoplasmic reticulum (ER) fragments, or ruptured lysosomes, preventing massive release of mitochondrial reactive oxygen species (mtROS), ER stress, or inflammatory activation. Dysregulation of this clearance mechanism may lead to sarcopenia, neuromuscular junction degeneration, and other pathological changes. Therefore, deciphering the receptors and molecular mechanisms of each organelle autophagy is a prerequisite for understanding muscle physiology and pathology and for targeting myopathy therapies. This section systematically describes the receptors and molecular mechanisms of six organelle autophagy types, laying a foundation for subsequent discussion of their roles in various skeletal muscle physiological and pathological contexts. It should be noted that this section introduces basic molecular mechanisms of organelle-specific autophagy, cited only as background knowledge, and thus was not subjected to the systematic search of this scoping review.

### 2.1 Mitophagy

Mitochondria are highly dynamic organelles in eukaryotic cells, consisting of an outer membrane (OM), inner membrane (IM), and intermembrane space (IMS) (Liu et al., 2025). As centers of Adenosine Triphosphate (ATP) production, mitochondria participate in regulating calcium ion (Ca^2+^), inflammatory cytokines, and reactive oxygen species (ROS) levels (Green and Kroemer, 2004), and maintain cellular homeostasis through the mitochondrial quality control (MQC) system (Ma and Ding, 2024). The MQC system includes mitochondrial biogenesis centered on PGC-1α/NRF/TFAM, mitochondrial dynamics centered on fission proteins Dynamin 1 Like (DNM1L)/Drp1, MFF, Fis1, and fusion proteins Mitofusin 1 (MFN1), Mitofusin 2 (MFN2), OPA1, and the mitophagy process (Liu et al., 2025; Zhou et al., 2025). As the core of MQC, mitophagy is tightly coupled with mitochondrial dynamics; mitochondrial fission is a prerequisite for mitophagy, and abnormal fusion also affects the mitophagy process (Ni et al., 2015). Moreover, mitophagy coordinates with mitochondrial biogenesis and is associated with the opening of the mitochondrial permeability transition pore (mPTP), jointly regulating cell fate (Liu et al., 2023).

**Figure 1:**
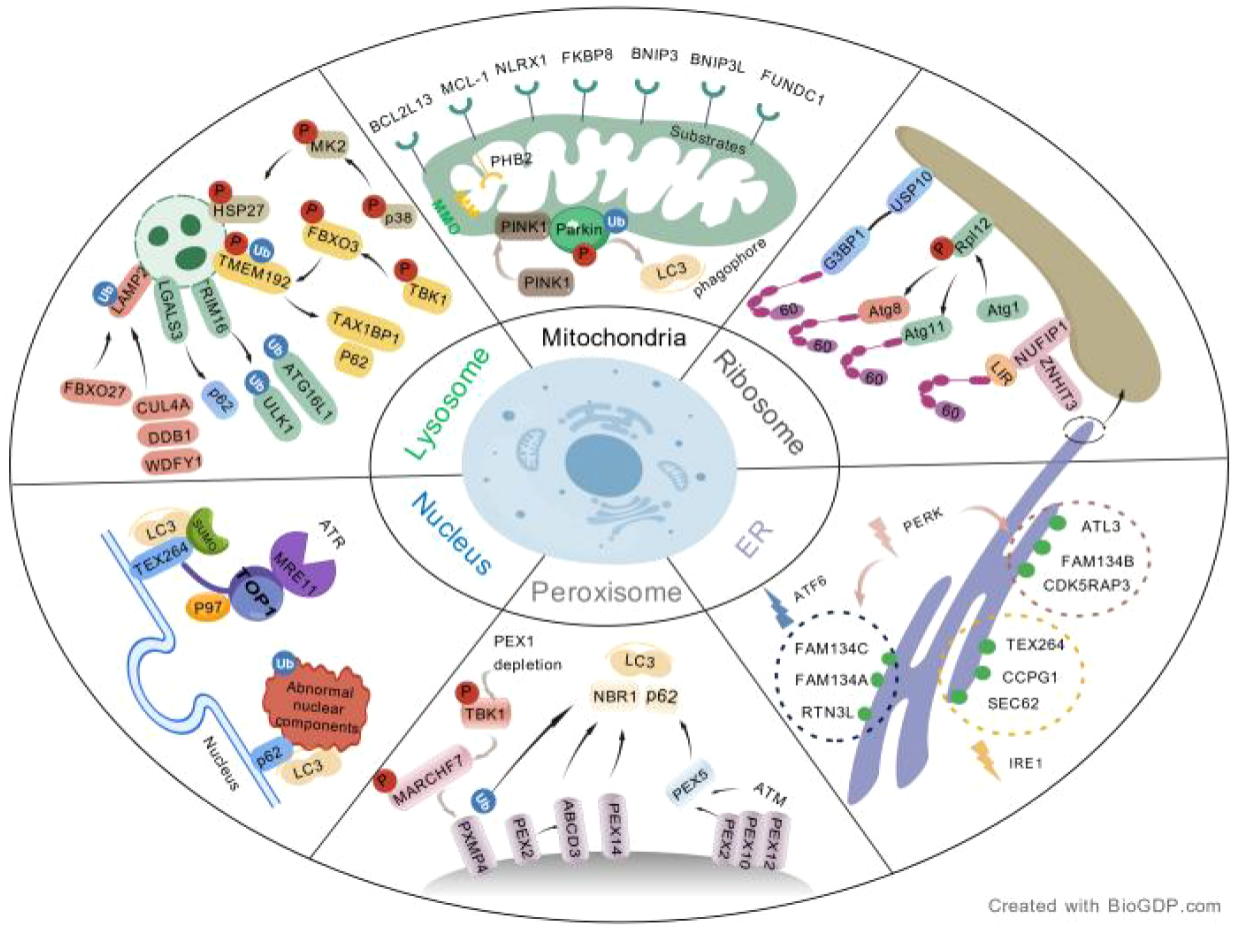
This figure systematically integrates the key regulatory pathways of six organelle‑specific autophagy processes.Mitophagy is primarily mediated by the PINK1‑Parkin ubiquitination pathway as well as receptors such as BNIP3, BNIP3L, FUNDC1, and BCL2L13; Lysophagy relies on core receptors including p62 and TAX1BP1; Ribophagy is mediated by molecules such as NUFIP1 and Rpl12; ER‑phagy under the regulation of ER stress signals (PERK, ATF6, IRE1), is mediated by receptors including FAM134B, ATL3, and TEX264 to clear damaged ER; Pexophagy selectively removes abnormal peroxisomes via PEX family proteins and receptors such as NBR1 and p62; Nucleophagy degrades aberrant nuclear components through molecules including TEX264 and p62. In the figure, Ub, P, and Small Ubiquitin-like Modifier (SUMO) represent ubiquitination, phosphorylation, and SUMOylation, respectively.

Mitophagy refers to a protective mechanism that selectively removes damaged or aged mitochondria under hypoxia and ROS accumulation stress, involving decreased mitochondrial membrane potential, autophagosome engulfment, and lysosomal degradation (Youle and Narendra, 2011). Mitophagy is mainly divided into ubiquitin-dependent and ubiquitin-independent pathways (see **Figure 1**). The ubiquitin-dependent pathway is represented by the PINK1-Parkin axis. Under basal conditions, PTEN-induced putative kinase 1 (PINK1) is translocated from the OM to the IM by translocases of the OM, then rapidly cleaved by the IM protease presenilin-associated rhomboid-like protein (PARL) and released back into the cytosol for proteasomal degradation, thereby maintaining low PINK1 levels on healthy mitochondria and preventing Parkin recruitment and activation on the OM, thus inhibiting mitophagy initiation (Deas et al., 2011; Meissner et al., 2011). Upon mitochondrial damage, PINK1 transport is blocked, leading to its accumulation on the OM, where it recruits and phosphorylates ubiquitin to activate Parkin (Narendra et al., 2010; Kane et al., 2014; Koyano et al., 2014). Beyond the classic PINK1-Parkin pathway, other ubiquitin-dependent mitophagy initiation mechanisms exist. For example, receptors such as Calcium binding and coiled-coil domain 2, also known as NDP52 (CALCOCO2) and Optineurin (OPTN) can directly recruit autophagy-related proteins (Yamano and Youle, 2020). Additionally, several E3 ubiquitin ligases, including SIAH1, Mitochondrial E3 ubiquitin protein ligase 1 (MUL1), and Ariadne RBR E3 ubiquitin protein ligase 1 (ARIH1), can mediate OM protein ubiquitination to trigger mitophagy. Among them, ARIH1 acts mainly dependent on PINK1, whereas MUL1 can be activated independently of PINK1 under certain stress conditions (Yun et al., 2014; Szargel et al., 2016; Villa et al., 2017). Notably, these alternative pathways may compensate when the PINK1-Parkin axis is impaired, highlighting the functional redundancy and context dependency of mitophagy regulation. The ubiquitin-independent pathway is mediated by receptors located on the OM, IM, or transmembrane proteins. These receptors directly initiate mitophagy by binding to LC3/GABA type A receptor-associated protein (GABARAP) proteins on the autophagosomal membrane via their LC3-interacting region (LIR) motifs (Antico et al., 2025). Notably, cross-talk exists between receptor-mediated mitophagy and the PINK1-Parkin pathway. For instance, BCL2 interacting protein 3 (BNIP3) and BNIP3L/NIX can form homodimers to act synergistically and are regulated by PRKN (Lee et al., 2011; Gao et al., 2015). Moreover, AMBRA1 can initiate the process either dependent on or independent of Parkin (PRKN) (Strappazzon et al., 2015; Gambarotto et al., 2022). Similarly, PHB2 mainly relies on PRKN for activation (Sun et al., 2025). Thus, understanding mitophagy requires moving beyond the PINK1-Parkin pathway to encompass a network of multiple receptor-dependent regulations.

### 2.2 ER-phagy

The ER is an extensive membrane system composed of flattened sheets and dynamic tubules, playing critical roles in protein quality control, lipid synthesis, Ca^2+^ homeostasis, and inter-organelle communication (Phillips and Voeltz, 2016). To maintain its functional homeostasis, cells have evolved a sophisticated ER quality control (ERQC) system, mainly comprising the unfolded protein response (UPR), ER-associated degradation (ERAD), and ER-phagy (Guan et al., 2024). When cells encounter stimuli such as nutrient deprivation, oxidative stress, or Ca^2+^ imbalance, the protein folding capacity of the ER is impaired, leading to the accumulation of unfolded or misfolded proteins and consequent ER stress. In response to this crisis, cells rapidly activate the UPR and ERAD pathways (Di Conza et al., 2023) to sequester potentially toxic unfolded proteins to specific regions. When misfolded protein accumulation exceeds ER degradation capacity, the highly selective ER-phagy process is activated (Yao et al., 2023). This process mainly relies on a series of specific ER-phagy receptors, which can be roughly classified into RHD-containing transmembrane receptors, Rh blood group D antigen (RHD)-lacking transmembrane receptors, and soluble receptors (see **Figure 1**) (Wilson and McCormick, 2025).

The Family with sequence similarity 134, also known as RETREG1 (FAM134) family comprises RHD-containing transmembrane receptors. FAM134B is activated by acetylation and phosphorylation upon ER stress, driving ER membrane curvature and fragmentation through oligomerization, and mediating degradation of ER fragments via its LIR domain binding to autophagosomes. FAM134B has two transcripts: the long isoform FAM134B-1 is regulated by extracellular ATP, whereas starvation-induced FAM134B-2 lacks part of the RHD domain but retains the TM3–4 transmembrane region and LIR motif (Keles et al., 2020); its function is not bulk ER degradation but rather selective autophagy of ER-retained secretory proteins, thereby maintaining amino acid homeostasis under nutrient deprivation (Kohno et al., 2019). Unlike FAM134B (Foronda et al., 2023), FAM134C is specifically distributed at ER tubules and sheet edges and responds to nutrient deprivation (Kumar et al., 2021); its oligomerization markedly induces fragmentation of ER subdomains and lysosomal delivery, but it induces ER fragmentation more slowly than FAM134B (Wilson and McCormick, 2025). Moreover, FAM134C exhibits low basal activity and requires activating signals for full activation, distinguishing it from FAM134B, which is fully active under basal conditions. Another family member, FAM134A, can function in an LIR-independent manner and compensate for the loss of FAM134B and FAM134C, respectively (Reggio et al., 2021). In addition to the FAM134 family, the long isoform of reticulon 3 (RTN3L) also contains an RHD, but unlike FAM134B, RTN3L harbors six LIR motifs within its long N-terminal region and preferentially binds GABARAP-L1; this interaction is completely lost only when all six LIR motifs are mutated (Grumati et al., 2017). Under nutrient-rich conditions, RTN3L participates in membrane trafficking via interaction with Rab9a; upon nutrient depletion, it switches to mediating ER tubule fragmentation to initiate ER-phagy (Wu and Voeltz, 2021).

Atlastin 3 (ATL3), SEC62 homolog, preprotein translocation factor (SEC62), Cell cycle progression 1 (CCPG1), and Testis expressed 264 (TEX264) are RHD-lacking transmembrane ER-phagy receptors that selectively mediate clearance of ER subdomains through distinct mechanisms. Among them, ATL3, localized to the tubular ER, specifically binds GABARAP via its GABARAP-interacting motifs (GIMs), thereby compensating for reduced degradation of tubular ER fragments caused by RTN3L deficiency (Chen et al., 2019b; Chen et al., 2019a). Additionally, SEC62 is a double-transmembrane protein whose upregulation enables direct binding to LC3, driving piecemeal micro-ER-phagy mediated by Endosomal Sorting Complexes Required for Transport III (ESCRT-III)/VPS4A independently of canonical autophagosomes, thereby delivering ER vesicles directly to lysosomes for degradation and effectively restoring ER homeostasis (Fumagalli et al., 2016; Loi et al., 2019). Meanwhile, CCPG1, a type II transmembrane protein localized at perinuclear ER (Smith and Wilkinson, 2018a; Smith et al., 2018), not only connects with autophagy proteins via its LIR domain but also interacts with the autophagy initiation complex RB1-inducible coiled-coil 1, also known as FIP200 (RB1CC1)/FIP200 through its unique RB1CC1/FAK family kinase-interacting protein of 200 kDa (FIP200)-interacting region (Smith and Wilkinson, 2018b; Zhou et al., 2021); its multiple cargo-interacting regions at the carboxyl terminus can specifically recognize and clear various luminal substrates (Ishii et al., 2023). TEX264, a type I transmembrane protein (Delorme-Axford et al., 2019; Fielden et al., 2022), is enriched at ER three-way junctions and functions not only under basal conditions but also mediates approximately half of ER-phagy during starvation (Chino et al., 2022).

On the other hand, soluble receptors CDK5 regulatory subunit associated protein 3 (CDK5RAP3) and Calcium binding and coiled-coil domain 1 (CALCOCO1) function by being recruited to the ER membrane. CDK5RAP3 contains shuffled Autophagy related 8 (ATG8)-interacting motifs (sAIMs) and normally binds to the ubiquitin-like protein UFM1; when ER stress triggers Ubiquitin-fold modifier 1 (UFM1) modification of ribosomal proteins (the ufmylation process), CDK5RAP3 is released and instead binds ATG8/GABARAP, thereby initiating autophagy (Walczak et al., 2019; Stephani et al., 2020; Picchianti et al., 2023). CALCOCO1 interacts with ER membrane proteins VAMP associated protein A and B (VAPA/B) via its C-terminal FFAT motif, two phenylalanines (FF) in an acidic tract (FFAT)-like motif and binds Atg8 family proteins through its LIR and UIR motifs; upon stress or starvation, it is recruited to the ER and promotes ER membrane morphological changes and fragmentation via oligomerization, thereby driving autophagosome formation (Nthiga et al., 2020b; Nthiga et al., 2020a).

### 2.3 Ribophagy

Ribosomes undertake nearly half of cellular protein translation (Warner, 1999); their biogenesis and protein translation processes are highly energy-intensive and tightly regulated by the cell. Under nutrient deprivation, increased ribosome degradation and downregulated protein synthesis are crucial for cell survival (Warner, 1999; Lafontaine, 2010). Ribophagy, a selective form of autophagy, is a key process for ribosome quality control (see **Figure 1**) (Kraft et al., 2008). In yeast, the ubiquitin-specific protease Ubiquitin-specific protease 3 (Ubp3) and its cofactor Bre5 protein, yeast homolog of human USP10 cofactor (Bre5) participate in ribophagy by regulating the 60S ribosomal subunit (Kraft and Peter, 2008; Kraft et al., 2008; Beese et al., 2020). This process also involves the E3 ligase Rsp5 and the Ubp3-Bre5 binding partners Cdc48 and Doa1/Ufd3 (Kraft and Peter, 2008; Ossareh-Nazari et al., 2010).

The discovery of the mammalian homologs of Ubp3-Bre5, USP10, and G3BP1 supports the conservation of the ribophagy pathway (Soncini et al., 2001). Similarly, the ribophagy-specific receptor Ribosomal protein L12 (Rpl12), conserved from yeast to mammals, is phosphorylated by Atg1 in yeast to enhance interaction with Atg11, promoting autophagosome assembly site formation; in mammals, Rpl12 binds Atg8/LC3 to mediate targeted degradation of the 60S ribosomal subunit (Chen et al., 2025; Tutak and Karbstein, 2025). In mammals under starvation or mechanistic target of rapamycin complex 1 (mTORC1) inhibition, Nuclear fragile X mental retardation protein interacting protein 1 (NUFIP1) acts as a key ribophagy receptor, directly binding Microtubule-associated protein 1 light chain 3 beta (MAP1LC3B) via its LIR motif to specifically mediate autophagic degradation of the 60S ribosomal subunit, thereby maintaining nucleotide and amino acid homeostasis (Denton and Kumar, 2018; Jin and Klionsky, 2018; Wyant et al., 2018); this process depends on the NUFIP1-interacting protein ZNHIT3 (Wyant et al., 2018).

### 2.4 Lysophagy

Lysosomes serve as the digestive apparatus of the cell, degrading abnormal intracellular components; however, their rupture leads to content leakage, damaging cells or even causing death. Under injury conditions, lysosomes themselves can become targets of autophagy, a process termed lysophagy, which is an important mechanism protecting cells against lysosomal damage (see **Figure 1**) (Hung et al., 2013; Maejima et al., 2013).

During acute injury, ruptured lysosomes are first recognized by sensor proteins such as Galectin-3 and -8 (Papadopoulos and Meyer, 2017; Skowyra et al., 2018); subsequently, the ESCRT-III complex is preferentially recruited to the injury site for membrane repair. If repair fails, the lysophagy clearance program is initiated (Radulovic et al., 2018). Thereafter, E3 ubiquitin ligases including SKP1-CUL1-F-box protein complex with F-box protein FBXO27 (SCF^FBXO27), Tripartite motif-containing protein family (TRIM16), and LRSAM1 cooperate with the E2 ubiquitin-conjugating enzyme UBE2QL1 to catalyze formation of K48- and K63-linked polyubiquitin chains at the injury site, which recruit autophagy receptors SQSTM1/Sequestosome-1, SQSTM1 (p62), Tax1 binding protein 1 (TAX1BP1), and the ATPases Associated with diverse cellular Activities (AAA-ATPase) Valosin containing protein, also p97 (VCP/p97), jointly promoting autophagosome formation to clear damaged lysosomes (Koerver et al., 2019; Mizushima, 2019; Kravic et al., 2020). Similar to other forms of selective organelle autophagy, degradation of damaged lysosomes relies on the ubiquitin-p62-MAP1LC3B pathway (Hung et al., 2013; Maejima et al., 2013). Upon lysosomal damage, elevated intracellular ROS levels activate p38 MAPK and its downstream kinase MK2 in an mTOR-dependent manner. Phosphorylated MAPK-activated protein kinase 2 (MK2) then phosphorylates the small heat shock protein HSP27 at Ser15, Ser78, and Ser82. p-Heat shock protein 27 (HSP27) is specifically recruited to damaged lysosomes, directly interacts with the adaptor protein p62, promotes p62 oligomerization via its PB1 domain to form liquid condensates, which then recognize K63-linked polyubiquitin chains and recruit autophagy-related proteins to facilitate local autophagosome formation (Gallagher and Holzbaur, 2023a; Gallagher and Holzbaur, 2023b; Gallagher et al., 2024; Jiménez-Loygorri and Boya, 2024; Jiménez-Loygorri et al., 2024). Recent studies have also revealed a novel lysophagy-regulating axis involving TANK-binding kinase 1 (TBK1)-SCF[FBXO3]-TMEM192-TAX1BP1 (Park and Cho, 2025; Park et al., 2025). Furthermore, VCP/p97 is a critical regulator of lysophagy, participating in the process by extracting damaged lysosomes decorated with K48-linked ubiquitin chains. Interfering with VCP function, such as through R155H and Tyr805 mutations, leads to the accumulation of K48-linked ubiquitinated substrates, further confirming the importance of VCP in lysophagy under persistent lysosomal damage (Papadopoulos et al., 2017; Kravić et al., 2022; Bai et al., 2023; Chauhan and Patro, 2024; Klickstein et al., 2024).

During autophagosomal engulfment of damaged lysosomes, the internal acidity and proteolytic activity can be gradually restored (Maejima et al., 2013), possibly due to fusion with intact lysosomes. Meanwhile, β-galactosidase released from damaged lysosomes can be sensed by the lysophagy marker Galectin 3 (LGALS3)/GAL3 (galectin-3) (Maejima et al., 2013). Additionally, lysosomal membrane permeabilization and elevated ROS also recruit the atypical TRIM family E3 ligase TRIM16 and galectin-3 (LGALS3); these two factors initiate the lysophagy response by ubiquitinating autophagy-related molecules such as Unc-51 like autophagy activating kinase 1 (ULK1) and ATG16L1 (Chauhan et al., 2016; Fraiberg and Elazar, 2016; Chae et al., 2023; Wang et al., 2023). In the lysosomal damage response, distinct E3 ubiquitin ligases also initiate selective autophagy by modifying the damaged lysosomal membrane glycoprotein LAMP2. For example, FBXO27, a glycoprotein-specific F-box protein, recognizes exposed Lysosomal associated membrane protein 2 (LAMP2) on damaged lysosomal membranes and catalyzes predominantly K48-linked ubiquitination, thereby regulating the recruitment of the autophagic machinery to damaged lysosomes (Yoshida et al., 2017). Similarly, the E3 ubiquitin ligase complex CUL4A-DDB1-WDFY1 also performs K48-linked polyubiquitination of LAMP2 following lysosomal damage, likewise promoting clearance of damaged lysosomes (Teranishi et al., 2022).

### 2.5 Pexophagy

Peroxisomes are dynamic single-membrane organelles that perform key functions including lipid metabolism, ROS handling, and detoxification; their abundance is coregulated by biogenesis, division, and autophagy (Tripathi and Walker, 2016; Eberhart and Kovacs, 2018; Li et al., 2025). Aberrant upregulation of pexophagy is a major mechanism underlying peroxisome biogenesis disorders, accounting for 65% of cases (Nazarko, 2017). Mammalian pexophagy mainly depends on ubiquitination of peroxisomal proteins, followed by recognition by p62 and NBR1 autophagy cargo receptor (NBR1) and targeting to the autophagic pathway for degradation (see **Figure 1**) (Kim et al., 2008; Deosaran et al., 2013). As key receptors, overexpression of either NBR1 or p62 can independently induce peroxisome clustering and degradation (Meuer et al., 1987; Schönenberger et al., 2015; Werner et al., 2019; Kim et al., 2025), and their cooperation enhances pexophagy efficiency (Deosaran et al., 2013). HSPA9 acts as an upstream regulator of pexophagy; its loss elevates ROS, inducing mitochondria-dependent pexophagy that requires ATG5, ATG7, and p62 (Jo et al., 2020). Meanwhile, the Cys-*N*-degron pathway modifies the N-terminal cysteine of ACAD10 via oxidation and arginylation, generating a degradation signal recognized by p62, thereby coregulating pexophagy under basal and stress conditions (Shim et al., 2023).

Regulation of pexophagy involves integration of various stress signals through a common molecular hub: ubiquitination of peroxisomal membrane proteins. Under nutrient starvation, mTORC1 inhibition upregulates the E3 ligase PEX2, which ubiquitinates Peroxisomal biogenesis factor 5 (PEX5) and Peroxisomal membrane protein 70 (PMP70), targeting peroxisomes for NBR1-mediated autophagic degradation (Ma et al., 2011; El Magraoui et al., 2012; Nordgren et al., 2015; Subramani, 2015; Zhang et al., 2015; Sargent et al., 2016; Tripathi et al., 2016). Notably, the peroxisomal membrane protein PEX14 can directly interact with MAP1LC3B under nutrient starvation to mediate canonical pexophagy (Hara-Kuge and Fujiki, 2008), even though PEX14 lacks a canonical LIR motif, suggesting that NBR1 or p62 may facilitate this interaction by inducing conformational changes (Jiang et al., 2015; Daussy et al., 2021). Additionally, PEX14 forms a complex with tankyrases Tankyrase (TNKS)/TNKS2 and ATG9A to promote a noncanonical pexophagy pathway independent of enzymatic activity under amino acid starvation (Li et al., 2017). Studies in methylotrophic yeast have shown that PEX14 is the only protein in the peroxisomal translocon complex that participates in both biogenesis and selective degradation (Zutphen et al., 2008), making PEX14 a molecular link connecting peroxisome homeostasis and autophagy regulation. Overexpression of another peroxisomal membrane protein, PEX3, under starvation induces peroxisome ubiquitination and clustering, followed by degradation via NBR1-mediated selective autophagy. However, this process does not require ubiquitination of PEX3 itself, suggesting the existence of other endogenous ubiquitination targets (Yamashita et al., 2014). Further research found that the ubiquitin ligase Membrane associated ring-CH-type finger 5 (MARCH5) is targeted to peroxisomes via PEX19 and PEX3 and ubiquitinates the membrane protein PMP70 during pexophagy, thereby triggering subsequent degradation (Zheng et al., 2022). Unlike starvation signals, hypoxic stress activates a parallel but mechanistically distinct pathway: EPAS1/HIF-2α promotes localization and oligomerization of NBR1 and p62 on peroxisomes while suppressing PPARα-driven peroxisome biogenesis, thus reducing peroxisome numbers to adapt to hypoxia (Schönenberger and Kovacs, 2015; Schönenberger et al., 2015). Notably, although the upstream regulators differ, both starvation- and hypoxia-induced pexophagy converge on the requirement for ubiquitination of peroxisomal membrane proteins. Moreover, monoubiquitination of PEX5 at its N-terminal C11 site or impairment of its mechanosensitive recycling from the membrane—such as upon loss of the p97-UBXD8 complex, dysfunction of the AAA ATPase complex (PEX1/PEX6/PEX26), or PEX13 deficiency—can also trigger pexophagy (Subramani, 2015; Law et al., 2017; Nazarko, 2017; Demers et al., 2023; Montes et al., 2025).

Antagonizing these propexophagy signals, the VCP-Fas associated factor 2 (FAF2) complex and USP30 serve as key negative regulators that maintain peroxisome homeostasis by preventing excessive degradation. Mechanistically, VCP-FAF2 extracts ubiquitinated PMP70 and modulates the ubiquitination status of ATP binding cassette subfamily D member 3, also PMP70 (ABCD3), thereby inhibiting OPTN-mediated excessive pexophagy (Koyano et al., 2024; Koyano and Matsuda, 2025). Ubiquitin specific peptidase 30 (USP30), a deubiquitinating enzyme, counteracts PEX2 by deubiquitinating PEX5 and ABCD3, thus maintaining peroxisome homeostasis (Sargent et al., 2016; Marcassa et al., 2019). This antagonism between peroxisomal membrane protein-mediated ubiquitination and VCP-FAF2-mediated extraction establishes a ubiquitination–deubiquitination balance that determines peroxisome fate. When this balance is disrupted, such as by ROS accumulation-induced catalase inhibition that enhances PEX5 ubiquitination and NBR1 recruitment (Lee et al., 2018), pexophagy is pathologically amplified, leading to peroxisome loss and metabolic dysfunction.

### 2.6 Nucleophagy

The nucleus is the core for storage and regulation of genetic material, and its quality control system is essential for maintaining genome stability (Sontag et al., 2023; Cha, 2024). As a key mechanism for nuclear quality control in mammalian cells, nucleophagy maintains nuclear integrity by clearing damaged or excess nuclear material, thereby supporting cellular health and homeostasis (Papandreou and Tavernarakis, 2019; Zhao et al., 2021).

As a selective form of autophagy, nucleophagy operates mainly through two core mechanisms. Macroscopic nucleophagy, the predominant form of nucleophagy in mammalian cells, involves bulk engulfment and degradation of larger nuclear structures and is critical for clearing aberrant nuclear components resulting from mitotic errors or genomic instability (Papandreou and Tavernarakis, 2020). This process begins with the ULK1/2 complex sensing and integrating upstream signals such as nutrient deprivation and DNA damage (Mercer et al., 2009; Kim et al., 2011; Zhou et al., 2017), followed by activation of the class III PtdIns3K complex localized on the ER and other membrane structures, catalyzing the generation of the lipid signal PtdIns3P (Fan et al., 2011; Fracchiolla et al., 2020; Gong and Pan, 2024). This promotes nucleation and expansion of the phagophore membrane, forming a double-membrane structure termed the autophagosome that specifically encloses and sequesters nuclear components destined for degradation, which are then transported via microtubules to lysosomes. Mediated by the HOPS complex (Zhang et al., 2023) and SNARE proteins (Tang et al., 2021; Jahn et al., 2024; Wang et al., 2024), the autophagosome fuses with a lysosome to form an autolysosome, where the contents are ultimately degraded by acid hydrolases and the degradation products are recycled by the cell.

In addition to macroscopic nucleophagy, nucleophagy also operates through autophagy receptors (see **Figure 1**) that simultaneously recognize intranuclear damage signals and autophagosomal membrane proteins, mediating targeted, precise degradation of specific nuclear injury sites—a core mechanism for fine regulation of nuclear quality (Behrends et al., 2010; Luo et al., 2016). For instance, p62 binds ubiquitinated nuclear proteins as well as LC3, mediating selective autophagic clearance of these protein aggregates (Pankiv et al., 2007; Rello-Varona et al., 2012). TEX264, a key receptor, directly binds LC3 via its LIR and specifically recognizes and hooks DNA topoisomerase 1 (TOP1) cleavage complexes stabilized on DNA, delivering them to the autophagic pathway for clearance, thereby repairing DNA damage (Fielden et al., 2020; Lascaux et al., 2024). Notably, TEX264 is a bifunctional receptor involved in both ER-phagy and nucleophagy, suggesting that different organelle-specific autophagy pathways share core autophagy receptors.

### 2.7 Cross-talk among multiple organelles in selective autophagy

Various organelle-specific autophagy pathways do not operate in isolation but jointly maintain cellular homeostasis through close cross-talk and coordination. First, multiple autophagy pathways share core regulatory elements, forming the basis for cross-talk. For example, the AAA-ATPase VCP/p97 not only promotes damaged mitochondrial segregation by extracting mitochondrial fusion proteins, providing a foundation for mitophagy, but also participates in forming the ELDR complex to facilitate lysophagy by handling ubiquitinated proteins on damaged lysosomal membranes. However, loss of VCP/p97 paradoxically enhances pexophagy, indicating substrate- and context-dependent functions (Papadopoulos and Meyer, 2017; Lee et al., 2018; Bai et al., 2023; Li et al., 2025). As another shared regulatory node, the kinase TBK1 phosphorylates the mitophagy receptors OPTN and CALCOCO2 as well as the lysophagy receptor TAX1BP1 to enhance their binding to LC3, thereby simultaneously regulating the efficiency of mitophagy and lysophagy (Eapen et al., 2021; Shima et al., 2023; Park et al., 2025). The E3 ubiquitin ligase MARCH5 and the deubiquitinating enzyme USP30 are both localized to mitochondria and peroxisomes, exhibiting cross-regulation in the autophagic machinery of these two organelles (Ma et al., 2011; Schönenberger and Kovacs, 2015; Antico et al., 2025; Kim et al., 2025). MARCH5 provides basal ubiquitination signals for Parkin on mitochondria; after induction of mitophagy, it is retargeted to peroxisomes via PEX19/PEX3, where it mediates PMP70 ubiquitination and promotes mTOR inhibition-induced pexophagy (Ma et al., 2011; Platta and Erdmann, 2022), thus linking the degradation regulation of mitochondria and peroxisomes. USP30, in turn, suppresses PINK1-Parkin-mediated mitophagy through deubiquitination (Ganley, 2018; Marcassa et al., 2018); it also localizes to peroxisomes and inhibits their basal autophagy, likely by deubiquitinating ABCD3 and PEX5 to counteract the E3 ligase PEX2 and maintain peroxisome homeostasis (Riccio et al., 2019; Koyano and Matsuda, 2025). The existence of such shared hubs allows a single stress signal to simultaneously mobilize multiple autophagy pathways for coordinated responses.

Second, physical contact sites between organelles are key platforms for direct and efficient cross-talk, among which ER-mitochondria contact sites (MAMs) are the most intensively studied. MAMs are not only hubs for exchanging signaling molecules such as Ca^2+^ and lipids but also require structural and functional integrity for effective mitophagy and ER-mitochondria cross-talk (Shi et al., 2024). For example, under stress such as hypoxia, the mitophagy receptor FUNDC1 accumulates at MAMs, where it interacts with the ER membrane protein calnexin (CANX) and recruits the fission protein DNM1L, synergistically initiating mitochondrial fission and autophagy (Wu et al., 2016a; Wu et al., 2016b). The tethering protein MFN2, a substrate of PINK1, is abundantly localized at MAMs and has been shown to play a role in regulating ER stress (de Brito and Scorrano, 2008; Dorn et al., 2015; Zorzano et al., 2015). Additionally, BECN1 and PINK1 are recruited to MAMs, promoting autophagosome formation and thereby enhancing mitophagy (Green and Kroemer, 2004; Gelmetti et al., 2017). This tight physical connection ensures that when abnormal Ca^2+^ release occurs from the ER, mitochondria can rapidly sense it and initiate autophagic clearance; conversely, mitochondrial dysfunction also affects ER homeostasis via MAMs (Hamasaki et al., 2013).

Furthermore, different selective autophagy pathways share core autophagic machinery, such as the ULK complex, PtdIns3K complex, ATG proteins, and MAP1LC3/GABARAP family proteins, and are regulated by common upstream signals, including mTORC1 and AMP-activated protein kinase (AMPK) (Hamasaki et al., 2013); they may also cooperatively regulate cellular quality control through specific receptors. For instance, TEX264 mediates both ER-phagy and, upon DNA damage, acts as a nucleophagy receptor to clear TOP1-DNA crosslinks (Rello-Varona et al., 2012). CALCOCO1 not only binds ER membrane proteins VAPA/B via its FFAT-like motif to mediate ER-phagy, but also drives Golgiphagy by binding Golgi membrane proteins Zinc finger DHHC-type containing palmitoyltransferases (ZDHHC17/ZDHHC13) via its zDABM motif (Nthiga et al., 2021; Yamamoto, 2021); studies also suggest it may participate in the clearance of mitochondria and peroxisomes, yet its loss only partially reduces ER-phagy efficiency while enhancing nonselective autophagy (Stefely et al., 2020). Another mitophagy receptor, BNIP3L, is localized to both the ER and mitochondria and can modulate the interaction between these two organelles (Diwan et al., 2009; Tagaya and Arasaki, 2017). Recent studies show that BNIP3L can also independently localize to peroxisomes and drive their autophagy, a process dependent on BNIP3L’s LIR and dimerization capacity (Wilhelm et al., 2022).

The six types of organelle-specific autophagy described above recognize and eliminate damaged organelles via their respective specific receptors, forming a core network of cellular quality control. However, the functional manifestations, regulatory mechanisms, and pathological significance of these mechanisms in skeletal muscle remain to be systematically reviewed. The following section presents a scoping review focusing on the current state of research on organelle autophagy in skeletal muscle physiology and pathology.

## 3 Part II: scoping review of organelle-specific autophagy in skeletal muscle physiology and pathology

### 3.1 Methods

Following the Preferred Reporting Items for Systematic reviews and Meta-Analyses extension for Scoping Reviews (PRISMA-ScR) guidelines (Tricco et al., 2018) and the Joanna Briggs Institute framework (Peters et al., 2015), we adopted a Population–Concept–Context framework, including studies on healthy individuals and those with skeletal muscle diseases (Population), investigating the mechanisms, regulation, or functions of various organelle-specific autophagy in skeletal muscle physiology and pathology (Concept), encompassing human, mouse, and rat *in vivo* experiments or skeletal muscle cell lines (Context).

We excluded conference abstracts, commentaries, duplicate publications, articles without full text, studies exclusively involving nonskeletal muscle cells, and those not discussing organelle-specific autophagy.

#### 3.1.1 Literature search

The search strategy was developed and executed with assistance from two researchers (CXW and GHL). We searched PubMed, Embase, Web of Science, and Cochrane Library for English articles published from database inception to 21 March 2026. Search terms combined skeletal muscle with mitophagy, ER-phagy, pexophagy, ribophagy, lysophagy, and nucleophagy (full search algorithms are provided in **Supplementary File 1**).

#### 3.1.2 Study selection

Two authors (CSG, FXW) independently screened the literature in two stages: first, titles and abstracts were screened to exclude obviously irrelevant records; then, both authors independently read the full texts and discussed eligibility based on inclusion criteria, extracting relevant information from included studies. Disagreements between the two authors were resolved by consulting a third author (QL).

#### 3.1.3 Data extraction

A data extraction form was first drafted using a representative sample of studies and then refined to cover the full scope of included studies. Data extraction was performed by one author (CXW) for consistency and independently verified by three other authors (GHL, CSG, FXW) to ensure accuracy. The form included: species (human/mouse/rat/cell), autophagy type, health status (healthy/disease), intervention, receptor, conclusions, limitations, and level of evidence.

#### 3.1.4 Limitations assessment

We assessed whether studies performed the following: (A) dual detection of autophagy markers (LC3B II + p62), (B) lysosomal inhibitor blocking experiments, (C) transmission electron microscopy (TEM)/fluorescent probes (mRFP-GFP-LC3), (D) bidirectional verification of autophagy function, and (E) reversibility of interventions.

#### 3.1.5 Evidence level analysis

For human studies, the Oxford Centre for Evidence-Based Medicine (OCEBM) levels of evidence (2009 version) were used (Durieux et al., 2013); for animal studies, the SYRCLE Risk of Bias (RoB) tool was applied (Hooijmans et al., 2014); for cell studies, a self-established rating criteria was used, including: (1) whether TEM was used to confirm autophagosomes (gold standard); (2) whether lysosomal inhibitors were used to distinguish autophagosome accumulation from genuine flux enhancement; (3) whether gene knockout/knockdown was used to exclude nonspecific effects; (4) whether the model used skeletal muscle cells (good) or nonmuscle cell lines (poor); (5) whether multi-time-point dynamic autophagy detection was performed.

### 3.2 Results

#### 3.2.1 General study details

**Figure 2:**
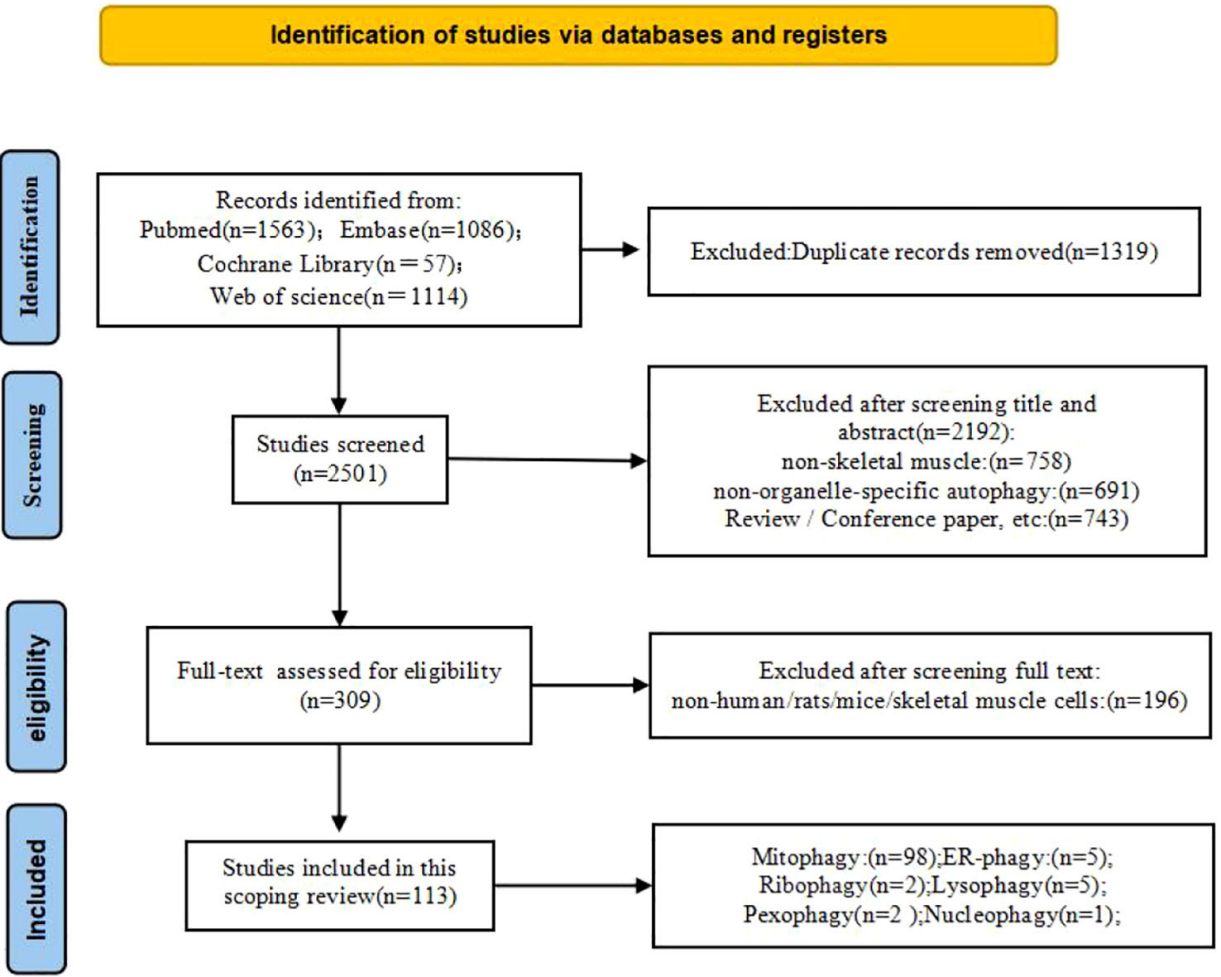
PRISMA flow chart.

A total of 3,820 articles were retrieved from the four selected databases. After removing duplicates, 2,501 titles and abstracts were screened, of which 309 proceeded to full-text review. Following screening and validation, 113 studies met all inclusion criteria and were retained for this scoping review. Selected information extracted from the included studies is summarized in **Figure 2**.

#### 3.2.2 Role of organelle-specific autophagy in skeletal muscle physiological processes

Building on the upstream molecular mechanisms, this section shifts focus to various organelle-specific autophagy types in skeletal muscle under physiological conditions, systematically reviewing how they transition from basal homeostasis maintenance to stress-adaptive responses in typical physiological states such as skeletal muscle development, exercise stress, nutritional/starvation stress, disuse atrophy, and physiological aging. See **Tables 1**–**5** for details.

##### 3.2.2.1 Exercise stress

**Table 1:** Organelle-specific autophagy under exercise stress.

Study	Species	Type	Physiology	Intervention	Receptor	Conclusion	Limitation	Evidence level
(Liu et al., 2025)	Rat	E	Health	Traditional Chinese medicine tuina	FAM134B	Tuina promotes FAM134B-mediated ER-phagy to facilitate skeletal muscle repair.	A:Y; B:N; C:Y; D:N; E:N	SYRCLE:L
(Jin et al., 2022)	Rat, L6 cell	E	Health	Acute exercise	FAM134B	FAM134B-mediated ER-phagy protects skeletal muscle in acute exercise stress.	A:LC3BII; B:N; C:Y; D:Y; E:Y	SYRCLE:M, cell: (1) Y; (2) N; (3)N; (4) Y; (5)Y
(Vainshtein et al., 2015b)	Mouse, C2C12 cell	M	Health	Acute exercise	BNIP3L, Parkin	Acute exercise simultaneously activates both mitochondrial biogenesis and mitophagy in skeletal muscle.	A:Y; B:Y; C:N; D:Y; E:N	SYRCLE:L, cell: (1) N; (2) Y; (3) Y; (4) Y; (5) Y
(Schwalm et al., 2017)	Human	M	Health	Acute high-intensity endurance exercise+different nutritional states	BNIP3, BNIP3L	Mitophagy is not activated within 1 h after acute endurance exercise, is mildly suppressed under fasting conditions, and is upregulated under the fed condition.	A:Y; B:N; C:N; D:N; E:Y	OCEBM:2b
(Dun et al., 2017)	Mouse	M	Health	Exhaustive exercise, aerobic exercise, rhodiola	BNIP3	Aerobic exercise combined with rhodiola ameliorates exhaustive exercise-induced skeletal muscle injury by regulating mitophagy.	A:Y; B:N; C:Y; D:N; E:N	SYRCLE:M
(Chen et al., 2018b)	Mouse	M	Health	Endurance exercise + exhaustive treadmill running	Parkin	Endurance training does not alter basal mitophagy but attenuates acute exercise-induced mitophagy.	A:Y; B:Y; C:N; D:N; E:N	SYRCLE:L
(Chen et al., 2018a)	Mouse	M	aging	A single bout of acute endurance exercise	Parkin	Acute exercise-induced mitophagy in skeletal muscle is strictly dependent on Parkin, and aging significantly attenuates this effect.	A:Y; B:Y; C:N; D:N; E:N	SYRCLE:M
(Arribat et al., 2019)	Human	M	Health	Moderate-intensity aerobic exercise	Parkin	Short-term exercise primarily induces mitochondrial fusion in skeletal muscle, whereas long-term exercise induces mitophagy and fusion while inhibiting fission.	A:Y; B:N; C:Y; D:N; E:N	OCEBM:3
(Balan et al., 2019)	Human	M	Health	Long-term endurance exercise	BNIP3, Parkin	Long-term endurance exercise maintains high expression of mitophagy, fission, and fusion proteins in skeletal muscle.	A:Y; B:N; C:N; D:N; E:N	OCEBM:4
(Hou et al., 2020)	Mouse	M	Health	Exhaustive exercise, rhodiola	PINK1, Parkin	Rhodiola suppresses PINK1-Parkin-mediated mitophagy and attenuates exhaustive exercise-induced structural damage to skeletal muscle mitochondria.	A:Y; B:N; C:Y; D:N; E:N	SYRCLE:M
(Triolo et al., 2022)	Mouse	M	Health	A single bout of exhaustive exercise	BNIP3, Parkin	Aging impairs mitochondrial clearance in skeletal muscle; females exhibit stronger basal clearance and a more pronounced compensatory response upon aging.	A:Y; B:N; C:N; D:N; E:N	SYRCLE:M
(Si et al., 2024)	Rat	M	Health	Exhaustive exercise, cannabidiol (CBD)	PINK1, Parkin, Bnip3	CBD ameliorates skeletal muscle by inhibiting excessive mitophagy induced by exhaustive exercise.	A:Y; B:N; C:Y; D:N; E:N	SYRCLE:M
(Shang et al., 2025)	Rat	M	Health	A single bout of acute eccentric exercise	FKBP8	Acute downhill running clears damaged mitochondria by inducing mitophagy.	A:LC3BII; B:N; C:Y; D:Y; E:N	SYRCLE:M

##### 3.2.2.2 Skeletal muscle development and regeneration

**Table 2:** Organelle-specific autophagy in skeletal muscle development and regeneration.

Study	Species	Type	Physiology	Intervention	Receptor	Conclusion	Limitation	Evidence level
(Buonomo et al., 2025)	C2C12 cell	E	Myogenesis	FAM134B1/2 KO	FAM134B1/2	FAM134B2 promotes ER remodeling and ER-phagy, thereby maintaining ER homeostasis in muscle cells and ensuring normal myotube maturation.	A:Y; B:Y; C:Y; D:Y; E:Y	Cell: (1) Y; (2) Y; (3) Y; (4) Y; (5) Y
(Cairns et al., 2024)	Mouse	M	Muscle regeneration	Pink1 KO	PINK1, Parkin	PINK1 maintains the self-renewal capacity of muscle stem cells by regulating mitophagy.	A:LC3BII; B:Y; C:Y; D:Y; E:N	SYRCLE:M
(Jiang et al., 2025)	Mouse, C2C12 cell	M	Myogenic differentiation and regeneration	Drp1KD	PINK1	Drp1 regulates myogenic differentiation and regeneration of skeletal muscle by mediating mitophagy.	A:Y; B:Y; C:Y; D:Y; E:Y	SYRCLE:M cell:①Y; ②Y; ③Y; ④Y; ⑤Y
(Rahman et al., 2025c)	Mouse	M	Myogenesis	MG132	BNIP3, BNIP3L, PINK1, Parkin	Mitophagy promotes skeletal muscle growth by clearing damaged mitochondria.	A:Y; B:N; C:N; D:Y; E:N	SYRCLE:H
(Rahman et al., 2025a)	C2C12 cell	M	Myogenic differentiation	DNM1L/Bnip3 KD/OE	BNIP3, BNIP3L	Controlled mitochondrial fission can initiate effective mitophagy and mitochondrial biogenesis; enhancing either autophagy or biogenesis alone cannot rescue myogenic disorders caused by fission defects.	A:Y; B:N; C:Y; D:Y; E:Y	Cell: (1) Y; (2) N; (3) Y; (4) Y; (5) Y
(Wang et al., 2026)	C2C12 cell	M	Myogenic differentiation	–	PINK1, Parkin, BNIP3	Mitophagy, mitocytosis, and apoptosis are activated sequentially and synergistically enhance mitochondrial clearance to promote myogenesis.	A:Y; B:N; C:Y; D:Y; E:Y	Cell: (1) Y; (2) N; (3) Y; (4) Y; (5) Y

##### 3.2.2.3 Nutrition/starvation stress

**Table 3:** Organelle-specific autophagy under nutrition/starvation stress.

Study	Species	Type	Physiology	Intervention	Receptor	Conclusion	Limitation	Evidence level
(Tarpey et al., 2017)	Human	M	Health	A single high-fat meal	Pink1, Parkin	A high-fat meal did not significantly alter mitophagy proteins in skeletal muscle of either group. In endurance-trained individuals, mitophagy and mitochondrial dynamics proteins in skeletal muscle were higher than in sedentary individuals under both fasting and postprandial conditions.	A:LC3BII; B:N; C:N; D:N; E:Y	OCEBM:4
(Fu et al., 2018)	Mouse, primary skeletal muscle cell	M	Obesity	High-fat meal	FUNDC1	FUNDC1 deficiency in skeletal muscle impairs mitophagy and reduces muscle function and endurance but upregulates FGF21 to promote white adipose tissue thermogenesis, ultimately conferring antiobesity effects and improving glucose metabolism under a high-fat diet.	A:Y; B:Y; C:Y; D:Y; E:N	SYRCLE:M, cell: (1) Y; (2) Y; (3) Y; (4) Y; (5) Y
(Heo et al., 2021)	Mouse	M	Obesity	High-fat meal	PINK1	Moderate-intensity aerobic exercise alleviates obesity-induced skeletal muscle mitochondrial damage and insulin resistance by restoring mitochondrial dynamics balance and attenuating overactivated mitophagy.	A:LC3BII; B:N; C:Y; D:N; E:N	SYRCLE:L
(Zheng et al., 2023)	Mouse	M	Hypercholesterolemia overload	High-fat meal	PINK1	Low-dose atorvastatin improves high-fat diet-induced skeletal muscle mitochondrial quality, morphology, and dysfunction by inhibiting mitophagy and enhancing mitochondrial fusion.	A:LC3BII; B:N; C:Y; D:N; E:N	SYRCLE:M
(Stouth et al., 2024)	Human, mouse	M	Fasting	Skeletal muscle-specific CARM1 KO	PRKN, Parkin, BNIP3	CARM1 delays fasting-induced skeletal muscle atrophy by regulating mitophagy.	A:Y; B:Y; C:Y; D:N; E:Y	OCEBM:4, SYRCLE:M

##### 3.2.2.4 Muscle disuse atrophy

**Table 4:** Organelle-specific autophagy under muscle disuse atrophy.

Study	Species	Type	Physiology	Intervention	Receptor	Conclusion	Limitation	Evidence level
(Figueiredo et al., 2021)	Human, rat	R	Muscle regeneration	Immobilization followed by reambulation	NUFIP1	Weight-bearing and resistance training reverse the enhanced ribophagy and reduced biosynthesis induced by disuse.	A:N; B:N; C:N; D:N; E:Y	OCEBM:4, SYRCLE:M
(Kotani et al., 2022)	Mouse	R	Muscle regeneration	Transcutaneous electrical nerve stimulation (pEMS)	NUFIP1	Peripheral electrical muscle stimulation (pEMS) alleviates disuse muscle atrophy by promoting ribosome synthesis and attenuating ribophagy.	A:LC3BII; B:N; C:N; D:N; E:Y	SYRCLE:M
(Drummond et al., 2014)	Human	M	Sedentary state with frailty	–	BNIP3, PINK1-Parkin	The expression of mitophagy receptors is downregulated in the skeletal muscle of elderly women with prolonged inactivity and frailty.	A:N; B:N; C:N; D:N; E:N	OCEBM:4
(Vainshtein et al., 2015a)	Mouse, C2C12 cell	M	Muscle regeneration	Unilateral sciatic nerve transection	BNIP3L	Peroxisome proliferator-activated receptor Peroxisome proliferator-activated receptor gamma coactivator 1-alpha (PGC-1α) regulates mitochondrial biogenesis and mitophagy, maintaining skeletal muscle adaptive regulation to chronic disuse.	A:Y; B:Y; C:Y; D:Y; E:N	SYRCLE:L, cell: (1) Y; (2) Y; (3) Y; (4) Y; (5) N
(Kang and Ji, 2016)	Mouse, C2C12 cell	M	Muscle regeneration	Immobilization followed by reambulation	PINK1, Parkin	Overexpression of PGC-1α inhibits excessive activation of the mitophagy pathway in skeletal muscle disuse atrophy, reducing mitochondrial ubiquitination and MFN2 degradation.	A:LC3BII; B:N; C:N; D:N; E:Y	SYRCLE:M, cell: (1) N; (2) N; (3) Y; (4) Y; (5) Y
(Kang et al., 2016)	Mouse	M	Muscle regeneration	Immobilization followed by reambulation	PINK1, Parkin, BNIP3	Partial mitophagy proteins remain elevated from the end of intervention up to 1 week later, and mitochondrial density and mtDNA are not fully restored.	A:Y; B:N; C:Y; D:N; E:Y	SYRCLE:M
(Graham et al., 2018)	Mouse	M	Muscle regeneration	Sciatic nerve transection	PINK1	After denervation, mitochondrial fusion decreases, fission increases, and mitophagy proteins are persistently upregulated in skeletal muscle.	A:Y; B:N; C:N; D:N; E:N	SYRCLE:H
(Leermakers et al., 2019)	Human, mouse	M	Muscle regeneration	immobilization	BNIP3, BNIP3L, FUNDC1, PINK1, Parkin	In the early phase of skeletal muscle unloading (3 days in mice, 7 days in humans), mitophagy signaling is enhanced and mitochondrial biogenesis regulation is downregulated, and these changes precede the overt decline in mitochondrial content.	A:Y; B:N; C:N; D:N; E:Y	OCEBM:4SYRCLE:M
(Yang et al., 2020)	Mouse, C2C12cell	M	Muscle regeneration	Sciatic nerve transection	PINK1, Parkin	Denervation upregulates miR-142a-5p and inhibits MFN1, inducing mitophagy and apoptosis, thereby driving skeletal muscle atrophy.	A:LC3BII; B:N; C:Y; D:Y; E:N	SYRCLE:M, cell: (1) Y; (2) N; (3) Y; (4) Y; (5) Y
(Deval et al., 2020)	Rat	M	Muscle regeneration	Immobilization followed by reambulation	Parkin, BNIP3, BNIP3L, FUNDC1	During immobilization, the gastrocnemius (GA) primarily activates the Parkin-independent pathway, whereas during reambulation, the tibialis anterior (TA) activates both Parkin-dependent and independent pathways, accompanied by significant activation of the CASA pathway, suggesting that anatomical location and mechanical tension coregulate mitophagy.	A:Y; B:N; C:N; D:N; E:Y	SYRCLE:M
(Yamashita et al., 2021)	Mouse	M	Muscle regeneration	Immobilization	BNIP3, BNIP3L, Parkin	Mitophagy activity is enhanced in skeletal muscle (primarily soleus) to clear damaged mitochondria during disuse muscle atrophy.	A:N; B:N; C:Y; D:N; E:N	SYRCLE:M
(Wang et al., 2023)	Rat	M	Muscle regeneration	Immobilization	BNIP3	During the early phase of immobilization followed by reambulation, persistent overactivation of BNIP3-dependent mitophagy exacerbates immobilization-induced skeletal muscle atrophy.	A:LC3BII; B:N; C:N; D:N; E:Y	SYRCLE:H
(Noone et al., 2025)	Human	M	Muscle regeneration	60-day 6° head-down tilt bed rest	PINK1, Parkin, BNIP3	Sixty days of bed rest lead to decreased mitochondrial content, fusion, and respiratory function in skeletal muscle, but mitophagy proteins do not show significant changes.	A:LC3BII; B:N; C:N; D:N; E:N	OCEBM:1b
(Noh et al., 2024)	Rat	M	Muscle regeneration	Tibial compression overload	PINK1, Parkin	Mitophagy markers in the quadriceps muscle remain elevated at the later stage (56 days).	A:Y; B:N; C:N; D:N; E:N	SYRCLE:H
(Liu et al., 2025)	Mouse	M	Muscle regeneration	Sciatic nerve transection	BNIP3	Astragaloside IV inhibits denervation-induced overactivation of mitophagy and delays skeletal muscle atrophy.	A:LC3BII; B:N; C:Y; D:N; E:N	SYRCLE:M
(Rahman et al., 2025b)	Mouse	M	Muscle regeneration	Immobilization	BNIP3, FUNDC1	Mitophagy protects against immobilization-induced skeletal muscle atrophy by clearing dysfunctional mitochondria.	A:Y; B:Y; C:Y; D:Y; E:N	SYRCLE:L
(Zhang et al., 2026)	C2C12 cell	M	Muscle regeneration	Idebenone, rotenone	Pink1, Parkin	Idebenone significantly ameliorates rotenone-induced muscle cell injury by regulating mitophagy and mitochondrial biogenesis.	A:Y; B:N; C:Y; D:N; E:Y	Cell: (1) Y; (2) N; (3) N; (4) Y; (5) N

##### 3.2.2.5 Age-related physiological muscle decline

**Table 5:** Organelle-specific autophagy in age-related physiological muscle decline.

Study	Species	Type	Physiology	Intervention	Receptor	Conclusion	Limitation	Evidence level
(Coen et al., 2024)	Human	P	Aging-related sarcopenia	–	–	Mitophagy- and pexophagy-related gene expressions are positively correlated with better muscle mitochondrial function, physical performance, and muscle volume.	A:N; B:N; C:N; D:N; E:N	OCEBM:4
(Carter et al., 2018)	Rat	M	Aging-related sarcopenia	Chronic contractile activity	BNIP3, BNIP3L, Parkin	Chronic contractile activity reduces excessive mitophagy flux and improves lysosomal function in aging muscle.	A:Y; B:Y; C:Y; D:N; E:N	SYRCLE:L
(Andreani et al., 2018)	Mouse	M	Aging-related sarcopenia	Oral ubiquinol + treadmill	BNIP3L	Exercise alone induces mitochondrial damage and mitophagy, whereas coenzyme Q10 combined with exercise downregulates mitophagy and apoptosis and improves mitochondrial structure and function.	A:N; B:N; C:Y; D:N; E:N	SYRCLE:M
(Leduc-Gaudet et al., 2019)	Mouse	M	Aging-related sarcopenia	Parkin OE	PINK1, Parkin	Parkin OE attenuates aging-related declines in muscle mass and strength by enhancing mitophagy and mitochondrial biogenesis.	A:N; B:N; C:Y; D:N; E:N	SYRCLE:L
(Shang et al., 2019)	Mouse	M	Aging-related sarcopenia	Parkin OE	Parkin	Parkin OE attenuates aging-related declines in muscle mass and strength by enhancing mitophagy and mitochondrial biogenesis.	A:Y; B:N; C:Y; D:N; E:N	SYRCLE:L
(Wang et al., 2020)	Mouse	M	Aging-related sarcopenia	Apigenin gavage	BNIP3	Apigenin alleviates aging-related sarcopenia by inhibiting mitophagy, reducing oxidative stress, and attenuating apoptosis.	A:Y; B:N; C:Y; D:N; E:N	SYRCLE:M
(Han et al., 2022)	Rat	M	Aging-related sarcopenia	MICT, HIIT	PINK1, Parkin	In the aging soleus muscle, high-intensity interval training (HIIT) upregulates mitochondrial biogenesis and mitophagy compared with moderate-intensity continuous training (MICT).	A:Y; B:N; C:N; D:N; E:N	SYRCLE:M
(Zhang et al., 2023)	Mouse, C2C12 cell	M	Aging-related sarcopenia	SPNS1 KO	PINK1, Parkin	SPNS1 deficiency impairs lysosomal function in skeletal muscle, thereby blocking mitophagy and ultimately leading to skeletal muscle atrophy and functional decline.	A:Y; B:N; C:Y; D:Y; E:N	SYRCLE:M, cell: (1) Y; (2) N; (3) Y; (4) Y; (5) N
(Zhou et al., 2025)	Mouse	M	Aging-related sarcopenia	PGC-1α OE	PINK1	Skeletal muscle-specific PGC-1α OE enhances mitophagy but does not restore the aging-induced decline in mitochondrial fusion proteins; instead, it reduces the expression of fission proteins.	A:N; B:N; C:N; D:N; E:N	SYRCLE:M
(Springer-Sapp et al., 2025b)	Rat	M	Aging-related sarcopenia	–	PINK1, Parkin	In the skeletal muscle of aging rats, mitochondrial content is significantly decreased, mitophagy protein expression is increased, whereas mitochondrial biogenesis markers show no significant change.	A:N; B:N; C:N; D:N; E:N	SYRCLE:M
(Cui et al., 2025)	Mouse, primary skeletal muscle mesenchymal stem cell	M	Aging-related sarcopenia	Colchicine, paclitaxel	BNIP3, BNIP3L, FUNDC1, PINK1, Parkin	Nanoplastics induce muscle cell aging through the microtubule–mitophagy–cGAS–STING axis, with Sirt2 being a key protective target.	A:N; B:N; C:Y; D:N; E:N	SYRCLE:H, cell: (1) Y; (2) N; (3) Y; (4) Y; (5) Y
(Di Lorenzo et al., 2025)	Rat	M	Aging-related sarcopenia	Short-term caloric restriction, resveratrol	PINK1, Parkin	Resveratrol upregulates mitochondrial fission and mitophagy in the skeletal muscle of aged rats, promoting clearance of damaged mitochondria.	A:N; B:N; C:N; D:N; E:N	SYRCLE:M
(Pérez-Guàrdia et al., 2025)	Mouse	M	Aging-related sarcopenia	–	PINK1	The reduction of STIM1 enhances mitophagy, accompanied by fast-to-slow muscle fiber transformation, delayed contraction/relaxation, and elevated muscle fatigability.	A:N; B:N; C:Y; D:N; E:N	SYRCLE:H

#### 3.2.3 Role of organelle-specific autophagy in skeletal muscle pathological processes

Building on the molecular mechanisms and physiological homeostasis regulation described above, this section further turns to organelle-specific autophagy in skeletal muscle under disease pathological conditions. By focusing on disease models that meet clinical diagnostic criteria or have clear pathological phenotypes, such as sarcopenia, diabetic myopathy, cancer cachexia, hereditary myopathies, inflammatory/immune-mediated myopathies, and other acquired myopathies, we systematically delineate the key transition and core roles of each organelle-specific autophagy from compensatory protective responses to pathological decompensatory damage. See **Tables 6**–**11** for details.

##### 3.2.3.1 Pathological sarcopenia

**Table 6:** Organelle-specific autophagy in pathological sarcopenia.

Study	Species	Type	Physiology	Intervention	Receptor	Conclusion	Limitation	Evidence level
(Xu et al., 2025)	Mouse, C2C12 cell	L	Sarcopenia	Glycerol, A-Au NPs	p62	The novel strategy of nanoimmunomodulation combined with macrophage delivery provides translational prospects for sarcopenia repair.	A:Y; B:N; C:Y; D:N; E:Y	SYRCLE:H, cell: (1) Y; (2) N; (3) N; (4) Y; (5) N
(Zhou et al., 2025)	Human	L	Sarcopenia	–	BHLHE41, HLTF, NPC1, UBE2D1/2, NDRG1, SLC22A18, CDKN1A, CALCOCO2, SCARB2, LGALS1, RPS27A	Lysophagy dysfunction contributes to the pathogenesis of sarcopenia by affecting mitochondrial function, energy metabolism, and immune cell infiltration.	A:N; B:N; C:N; D:N; E:N	OCEBM:2b
(Ghzaiel et al., 2022)	C2C12 cell	P	Sarcopenia	Milk thistle seed oil, α-tocopherol	Pex5, Pex13, Pex14	7β-Hydroxycholesterol induces pexophagy while suppressing peroxisome biogenesis in C2C12 myoblasts.	A:N; B:N; C:Y; D:N; E:Y	(1) Y; (2) N; (3) N; (4) Y; (5) N
(Sebastián et al., 2016)	Mouse, C2C12 cell	M	Sarcopenia	MFN2 KD/KO	BNIP3	In skeletal muscle, reduced MFN2 impairs mitophagy and activates the HIF1α-BNIP3 pathway via ROS, partially compensating for the mitophagy defect.	A:Y; B:Y; C:Y; D:Y; E:N	SYRCLE:M, cell: (1) Y; (2) Y; (3) Y; (4) Y; (5) Y
(Sakellariou et al., 2016)	Mouse	M	Sarcopenia	–	PINK1, Parkin	Mitophagy participates in maintaining mitochondrial integrity but is not a key driver of sarcopenia.	A:N; B:N; C:N; D:N; E:N	SYRCLE:H
(Springer-Sapp et al., 2025a)	Human	M	Sarcopenia	Lower limb resistance training	Parkin	MQC protein expression in mild-to-moderate sarcopenia shows no significant difference from controls; 12 weeks of resistance training increases muscle strength and MFN2 expression without significantly elevating mitophagy or MQC proteins.	A:N; B:N; C:N; D:N; E:N	OCEBM:2b
(Chen et al., 2025)	Mouse, C2C12 cell	M	Sarcopenia	Astragalus polysaccharides (APS)	PINK1, Parkin	APS alleviates skeletal muscle aging by stabilizing PINK1 protein and enhancing mitophagy.	A:LC3BII; B:N; C:N; D:Y; E:N	SYRCLE:H, cell: (1) N; (2) N; (3) Y; (4) Y; (5) Y
(Zhao et al., 2026)	Rat, C2C12 cell	M	Sarcopenia	Icariin	PINK1, Parkin	Icariin ameliorates muscle atrophy by inhibiting overactivated mitophagy in sarcopenia.	A:Y; B:N;C:N; D:N; E:N	SYRCLE:M, cell: (1) N; (2) N; (3) N; (4) Y; (5) N

##### 3.2.3.2 Diabetes-related muscle atrophy

**Table 7:** Organelle-specific autophagy in diabetes-related muscle atrophy.

Study	Species	Type	Pathology	Intervention	Receptor	Conclusion	Limitation	Evidence level
(Nguyen et al., 2025)	L6, C2C12, iPSC-derived human muscle cell, mouse	E	Insulin resistance	ALY688	FAM134B	FAM134B-dependent endoplasmic reticulum autophagy alleviates endoplasmic reticulum stress induced by iron overload, thereby improving insulin sensitivity	A:Y; B:Y; C:Y; D:N; E:N	SYRCLE:L, cell: (1) Y; (2) Y; (3) Y; (4) N; (5) N
(Brinkmann et al., 2017)	Human	M	Obese patients with T2DM	Endurance training	BNIP3	Mitochondrial autophagy proteins showed no significant changes, but the content of mitochondrial oxidative phosphorylation complex II was significantly increased.	A:LC3BII; B:N; C:N; D:N; E:N	OCEBM:4
(Chen et al., 2018)	L6 cell	M	Insulin resistance	Puerarin	PINK1, Parkin	Puerarin improves insulin sensitivity in skeletal muscle cells by promoting PINK1-Parkin-mediated mitochondrial autophagy.	A:Y; B:N; C:Y; D:N; E:N	Cell: (1) Y; (2) N; (3) N; (4) Y; (5) N
(Gundersen et al., 2020)	Primary human skeletal muscle myotube	M	Obesity + T2DM	–	Parkin, PINK1	Obese individuals exhibit highly fragmented skeletal muscle mitochondria, with significantly reduced Parkin levels in obese patients complicated by type 2 diabetes mellitus (T2DM).	A:LC3BII; B:N; C:N; D:N; E:N	Cell: (1) N; (2) N; (3) N; (4) Y; (5) N
(Ehrlicher et al., 2021)	Mouse, C2C12cell	M	Obesity + early-stage T2DM	High-fat diet	BNIP3	Short-term hyperlipidemia activates skeletal muscle mitochondrial autophagy without inducing significant changes in downstream autophagosomes and lysosomes.	A:Y; B:Y; C:N; D:N; E:Y	SYRCLE:M, cell: (1) N; (2) Y; (3) N; (4) Y; (5) Y
(Yan et al., 2023)	Mouse, C2C12cell	M	T2DM	STING KO	PINK1, Parkin	STING deficiency enhances PINK1-Parkin-mediated mitochondrial autophagy, alleviating insulin resistance and diabetes-associated muscle fiber atrophy	A:Y; B:N; C:Y; D:Y; E:N	SYRCLE:H, (1) Y; (2) N; (3) Y; (4) Y; (5) N
(Deng et al., 2024)	Rat	M	T1DM	Celecoxib	BNIP3	Celecoxib improves diabetic muscle atrophy by inhibiting endoplasmic reticulum stress and mitochondrial dysfunction.	A:LC3BII; B:N; C:Y; D:N; E:N	SYRCLE:H
(Zhao et al., 2024)	Mouse, C2C12cell	M	Diabetes mellitus	d-Chiral inositol	PINK1, Parkin	d-Chiral inositol alleviates diabetes-associated sarcopenia induced by mitochondrial autophagy defects through downregulation of MFG-E8	A:Y; B:N; C:Y; D:Y; E:N	SYRCLE:H, cell: (1) Y; (2) N; (3) Y; (4) Y; (5) N
(Moustafa Mahmoud et al., 2025)	Rat	M	T1DM	MIE, HIE + insulin	BNIP3, PINK1, Parkin	HIE can downregulate T1DM-induced hyperactivated mitochondrial autophagy and restore muscle mass and strength	A:Y; B:N; C:N; D:N; E:N	SYRCLE:M
(Dantas et al., 2025)	Human, C2C12cell	M	Obesity + T2DM	–	PINK1, Parkin	In patients with type 2 diabetes mellitus (T2DM), mitochondrial autophagy in skeletal muscle is inhibited, and mitochondrial quality control defects lead to increased mitochondrial fragmentation and decreased insulin sensitivity.	A:Y; B:N; C:Y; D:Y; E:N	OCEBM:4, cell: (1) Y; (2) N; (3) Y; (4) Y; (5) N
(Hoolachan et al., 2025)	Mouse, L6cell	M	T2DM	STX4 KO	PINK1, Parkin	STX4 maintains mitochondrial structural and functional homeostasis in skeletal muscle by regulating mitochondrial biogenesis and autophagy.	A:N; B:N; C:Y; D:N; E:N	SYRCLE:M, cell: (1) Y; (2) N; (3) Y; (4) Y; (5) N
(Xu et al., 2025)	Mouse, C2C12cell	M	Diabetic muscle	AA-sEV	PINK1	AA-sEV restores the balance between protein synthesis and degradation by activating PINK1-mediated mitochondrial autophagy, thereby alleviating diabetes-induced muscle atrophy.	A:LC3BII; B:N; C:Y; D:N; E:N	SYRCLE:M cell: (1) Y; (2) N; (3) Y; (4) Y; (5) N

##### 3.2.3.3 Cachexia-related muscle atrophy

**Table 8:** Organelle-specific autophagy in cachexia-related muscle atrophy.

Study	Species	Type	Pathology	Intervention	Receptor	Conclusion	Limitation	Evidence level
(Marzetti et al., 2017)	Human	M	Elderly patients with gastric cancer	–	PINK1, Parkin	In skeletal muscles of elderly gastric cancer patients, multiple dysregulations occur in mitochondrial dynamics, mitochondrial autophagy, and autophagic flux.	A:LC3BII; B:N; C:N; D:N; E:N	OCEBM:4
(Brown et al., 2017)	Mouse	M	Cancer cachexia	–	BNIP3, PINK1, Parkin	Early targeted protection of mitochondria can serve as a novel approach to prevent or delay cancer cachexia.	A:N; B:N; C:Y; D:N; E:N	SYRCLE:H
(Ballarò et al., 2019)	Mouse	M	Cancer cachexia	Moderate-intensity exercise	BNIP3, PINK1, Parkin	Regular endurance exercise promotes mitochondrial fission and autophagy in human skeletal muscle independent of age, maintaining mitochondrial quality homeostasis.	A:Y; B:N; C:N; D:N; E:N	SYRCLE:H
(Zhang et al., 2022)	Mouse, C2C12 myogenic cell	M	Cancer-related fatigue (CRF)	paclitaxel	BNIP3, PINK1, Parkin	Paclitaxel induces skeletal muscle mitochondrial autophagy through the PHD2-HIF-1α-BNIP3 signaling pathway, thereby alleviating cancer-related fatigue	A:Y; B:N; C:Y; D:Y; E:N	SYRCLE:H, cell: (1) Y; (2) N; (3) Y; (4) Y; (5) N
(Zhang et al., 2023)	Human, mouse	M	Cancer cachexia	Mitochondrial division inhibitor 1	BNIP3, PINK1	Overactivated mitochondrial autophagy is involved in the occurrence of skeletal muscle atrophy in cancer cachexia, while inflammatory responses and oxidative stress may be associated with this process.	A:LC3BII; B:N; C:Y; D:N; E:N	OCEBM:4, SYRCLE:H
(Guo et al., 2024)	Mouse, C2C12cell	M	CRF	Shenqi Fuzheng injection	PINK1	Shenqi Fuzheng injection alleviates chronic renal failure by promoting mitochondrial autophagy in skeletal muscle.	A:Y; B:N; C:Y; D:Y; E:N	SYRCLE:H, cell: (1) Y; (2) N; (3) Y; (4) Y; (5) N
(Zhang et al., 2024)	Mouse, C2C12 cells, and differentiated myotubes	M	Cancer cachexia	MG132, S100A9	BNIP3	The tumor-secreted S100A9 overactivates mitochondrial autophagy in skeletal muscle through its receptor AGER, leading to skeletal muscle atrophy.	A:Y; B:Y; C:Y; D:Y; E:N	SYRCLE:H, cell: (1) Y; (2) Y; (3) Y; (4) Y; (5) Y
(Fornelli et al., 2024)	Mouse	M	Cancer cachexia	BNIP3 KD	BNIP3	Knockdown of BNIP3 inhibits overactivated mitochondrial autophagy in cancer cachexia and delays myofibril atrophy	A:LC3BII; B:N; C:Y; D:N; E:N	SYRCLE:H
(Morena et al., 2025)	Mouse	M	Cancer cachexia	Bnip3 KO	BNIP3	Compared to males, female mice exhibited less tumor-induced mitochondrial dysfunction, and the deletion of BNIP3 did not affect basal protein synthesis or loss of muscle mass.	A:LC3BII; B:N; C:Y; D:N; E:N	SYRCLE:H
(Tian et al., 2025)	Mouse	M	Cancer cachexia	APS	BNIP3	APS reverses hyperactivated mitochondrial autophagy in cancer cachexia, leading to muscle atrophy and dysfunction	A:Y; B:N; C:Y; D:N; E:N	SYRCLE:M
(Wang et al., 2026)	Mouse, C2C12cell	M	Cancer cachexia	OH-CATH30	BNIP3	OH-CATH30 alleviates skeletal muscle atrophy by inhibiting TLR4 and downregulating the mitochondrial autophagy receptor BNIP3 protein.	A:LC3BII; B:N; C:N; D:N; E:N	SYRCLE:H, cell: (1) N; (2) N; (3) N; (4) Y; (5) Y

##### 3.2.3.4 Hereditary myopathies

**Table 9:** Organelle-specific autophagy in hereditary myopathies.

Study	Species	Type	Pathology	Intervention	Receptor	Conclusion	Limitation	Evidence level
(Park et al., 2009)	Human, mouse, mouse fibroblast	N	Nuclear envelope disease	–	Lamin A/C	Nuclear autophagy degrades damaged nuclear DNA, histone H1, γH2AX-marked damaged DNA, and nuclear particles.	A:LC3BII; B:Y; C:Y; D:N; E:N	OCEBM:4, SYRCLE:H, cell: (1) Y; (2) Y; (3) Y; (4) N; (5) N
(Mitsuhashi et al., 2011)	Mouse	M	Congenital muscular dystrophy	Cholinesterase β gene function KO/mutation	PINK1, Parkin	Cholinesterase deficiency overactivates mitochondrial autophagy, leading to a reduction in muscle mitochondrial quantity and pathological changes.	A:Y; B:N; C:Y; D:N; E:N	SYRCLE:H
(Arhzaouy et al., 2019)	Mouse, HeLa/U2OS cell	L	VCP KO	–	LGALS3	In the differentiation of skeletal muscle, VCP can coordinate the balance between lysosomal autophagy and lysosomal biogenesis.	A:Y; B:N; C:Y; D:N; E:N	SYRCLE:L, cell: (1) Y; (2) N; (3) Y; (4) N; (5) Y
(Zhu et al., 2022)	Mouse, nonskeletal muscle cell	L	Multisystem protein disorders associated with VCP gene mutations	VCP KO/OE and mutations	–	VCP clears damaged late endosomes/lysosomes through lysosomal autophagy, thereby inhibiting the protein aggregation and dissemination of α-synuclein and TDP-43.	A:N; B:N; C:N; D:N; E:N	SYRCLE:M, cell: (1) N; (2) N; (3) Y; (4) N; (5) N
(Borgia et al., 2017)	Human, C2C12 cell	M	SBMA/ALS/neurogenic atrophy	–	BNIP3, PINK1	Excessive activation of mitochondrial autophagy may be a key pathological mechanism in SBMA skeletal muscle	A:Y; B:N; C:Y; D:N; E:N	OCEBM:4, cell: (1) Y; (2) N; (3) N; (4) Y; (5) N
(Sato et al., 2018)	Mouse, primary skeletal muscle tubules	M	Congenital myopathy	Apobec2 KO	BNIP3	The Apobec2 deficiency induces abnormalities in the morphology and function of skeletal muscle mitochondria, subsequently triggering enhanced mitochondrial autophagy as a defensive response. Prolonged exposure leads to myopathic atrophy.	A:Y; B:N; C:Y; D:N; E:N	SYRCLE:H, cell: (1) Y; (2) N; (3) Y; (4) Y; (5) N
(Alshudukhi et al., 2018)	Mouse, primary skeletal muscle tubules	M	Hereditary rhabdomyolysis	Lipin-1 KO	Bnip3	A deficiency in lipin-1 impairs mitochondrial autophagy in glycolytic skeletal muscle, resulting in reduced muscle contractility, whereas oxidative muscle fibers are less affected.	A:Y; B:Y; C:Y; D:Y; E:N	SYRCLE:M, cell: (1) Y; (2) Y; (2) Y; (4) Y; (5) N
(Dubinin et al., 2022)	Mouse	M	Duchenne muscular dystrophy	Uridine	PINK1, Parkin	Uridine can upregulate Parkin gene expression in skeletal muscles of Duchenne muscular dystrophy mice but has no significant effect on Pink1 or mitochondrial DNA.	A:N; B:N; C:Y; D:N; E:N	SYRCLE:M
(Wu et al., 2023)	Mouse, C2C12 cell	M	Duchenne muscular dystrophy	AAV-TRIM72	BNIP3L	TRIM72 alleviates inflammatory responses in skeletal muscles of Duchenne muscular dystrophy mice by promoting mitochondrial autophagy and inhibiting the activation of NLRP3 inflammasome.	A:Y; B:Y; C:Y; D:Y; E:N	SYRCLE:L, cell: (1) Y; (2) Y; (3) Y; (4) Y; (5) N
(Li et al., 2025)	Mouse, C2C12 cell	M	Duchenne muscular dystrophy	l-NAME	PINK1, Parkin	The l-NAME component partially activates PINK1-Parkin-mediated mitochondrial autophagy, ameliorating necrosis in skeletal muscles of Duchenne muscular dystrophy mice.	A:LC3BII; B:N; C:Y; D:N; E:N	SYRCLE:H, cell: (1) Y; (2) N; (3) N; (4) Y; (5) N

##### 3.2.3.5 Inflammatory/immune-mediated myopathies

**Table 10:** Organelle-specific autophagy in inflammatory/immune-mediated myopathies.

Study	Species	Type	Physiology	Intervention	Receptor	Conclusion	Limitation	Evidence level
(Vittonatto et al., 2017)	Human	L	Sporadic inclusion body myositis	–	p62	P62 immunohistochemical positivity can serve as an auxiliary diagnostic marker for sporadic inclusion body myositis.	A:Y; B:N; C:N; D:N; E:N	OCEBM:4
(Ko et al., 2016)	Mouse	M	Inflammation + aging	IL-10 KO	Bnip3L	Chronic inflammation suppresses aging-induced upregulation of mitochondrial autophagy	A:LC3BII; B:N; C:Y; D:N; E:N	SYRCLE:H
(Matsubara et al., 2018)	Human	M	Antibodies against HMGCR-associated immunization	–	BNIP3	Mitochondrial autophagy plays a significant role in skeletal muscle fiber degeneration associated with anti-HMGCR antibody-mediated necrotizing myopathy	A:LC3BII; B:N; C:Y; D:N; E:N	OCEBM:5
(Leermakers et al., 2020)	Mouse	M	Acute lung injury and pulmonary inflammation	Single intratracheal drip of LPS	BNIP3, BNIP3L, FUNDC1, PINK1, Parkin	A single intratracheal infusion of LPS-induced acute pulmonary inflammation activates mitochondrial autophagy and transiently downregulates mitochondrial biogenesis in both oxidative and glycolytic skeletal muscles of mice, without causing a significant reduction in mitochondrial content.	A:Y; B:N; C:N; D:N; E:N	SYRCLE:M
(Zhang et al., 2024)	Rat	M	Autoimmune myasthenia gravis	AS-IV	PINK1, Parkin	ASIV improves autoimmune myasthenia gravis-induced gastrocnemius muscle injury in rats by upregulating PINK1-Parkin-mediated mitochondrial autophagy and inhibiting mitochondrial apoptosis.	A:Y; B:N; C:Y; D:N; E:N	SYRCLE:H
(Aggarwal et al., 2024)	Mouse	M	Infect	D88, SS-31	Parkin	D88/SS-31 reverses the inhibitory effect of the population-sensing transcription factor MvfR from *Pseudomonas aeruginosa* on skeletal muscle mitochondrial autophagy	A:LC3BII; B:N; C:N; D:N; E:Y	SYRCLE:H
(Han et al., 2024)	Mouse, primary muscle satellite cell	M	Inflammation	Tan IIA	BNIP3	Tan IIA alleviates inflammation-induced skeletal muscle atrophy by inhibiting mitochondrial hyperfission and mitochondrial autophagy.	A:Y; B:N; C:Y; D:N; E:N	SYRCLE:M, cell: (1) Y; (2) N; (3) N; (4) Y; (5) N
(Li et al., 2025)	Rat, C2C12 cell	M	Chewing muscle atrophy	NLRP3 inflammatory small body agonist NIA	PINK1, Parkin	NLRP3 inflammatory bodies induce mitochondrial dysfunction and overactivate PINK1-Parkin-mediated mitochondrial autophagy, leading to masticatory muscle atrophy.	A:LC3BII; B:N; C:Y; D:N; E:N	SYRCLE:L, cell: (1) Y; (2) N; (3) Y; (4) Y; (5) Y

##### 3.2.3.6 Other myopathies

**Table 11:** Organelle-specific autophagy in other myopathies.

Study	Species	Type	Physiology	Intervention	Receptor	Conclusion	Limitation	Evidence level
(Field et al., 2025)	Mouse, C2C12 cell	E	Ruptured red fiber-type mitochondrial myopathy	Downregulate BNIP3L electric pulse stimulation	BNIP3L	BNIP3L simultaneously promotes mitochondrial autophagy and endoplasmic reticulum autophagy, maintaining mitochondrial/endoplasmic reticulum homeostasis, muscle fiber type, and metabolic phenotype in skeletal muscle.	A:Y; B:N; C:Y; D:Y; E:N	SYRCLE:L, cell: (1) Y; (2) N; (3) Y; (4) Y; ⑤N
(White et al., 2016)	Human	M	Peripheral arterial disease of the lower extremities	–	PINK1, Parkin	Impaired fusion or degradation of autophagosomes with lysosomes may be associated with impaired muscle oxidation and gait function.	A:LC3BII; B:N; C:Y; D:N; E:N	OCEBM:4
(He et al., 2020)	Mouse, C2C12 cell	M	Critical limb ischemia model	Moderate-intensity treadmill exercise	PINK1, Parkin	Moderate-intensity exercise significantly upregulates mitochondrial autophagy in gastrocnemius muscles of aged lower limb ischemic mice and alleviates ischemic myopathy.	A:LC3BII; B:N; C:Y; D:N; E:N	SYRCLE:M, cell: (1) Y; (2) N; (3) Y; (4); (5) Y
(Jiang et al., 2024)	Rat	M	Acute compartment syndrome of the thigh	Injection of sodium octanoate during fasciotomy	PINK1, Parkin	Sodium caprylate alleviates skeletal muscle injury induced by acute compartment syndrome by promoting mitochondrial autophagy and improving mitochondrial dynamics.	Ap62; B:N; C:N; D:N; E:N	SYRCLE:M
(Zhang et al., 2019)	Human	M	Chronic kidney disease		BNIP3, PINK1, Parkin	Mitochondrial autophagy is hyperactivated and plays a significant role in skeletal muscle atrophy in patients with chronic kidney disease.	A:Y; B:N; C:Y; D:N; E:N	OCEBM:4
(Rogers et al., 2017)	Mouse	M	Amyotrophic lateral sclerosis	–	Bnip3, Pink1, Parkin	Pink1-Parkin-mediated mitochondrial autophagy dysfunction is a key mechanism underlying neuromuscular junction (NMJ) degeneration in amyotrophic lateral sclerosis (ALS).	A:LC3BII; B:N; C:Y; D:N; E:N	SYRCLE:H
(Leermakers et al., 2018)	Human	M	Clinically stable COPD	–	BNIP3, BNIP3l, FUNDC1, PINK1, Parkin	Enhanced mitochondrial autophagy signaling in skeletal muscle of COPD patients is associated with reduced mitochondrial content and disease severity.	A:LC3BII; B:N; C:N; D:N; E:N	OCEBM:4
(Mao et al., 2020)	Rat, L6 cell	M	COPD	Gastric administration of the Fufei Jianpi formula	PINK1, Parkin	The Fufei Jianpi formula promotes mitochondrial biogenesis in COPD rats and L6 cells, inhibits mitochondrial autophagy, and improves skeletal muscle mitochondrial function.	A:Y; B:N; C:Y; D:Y; E:N	SYRCLE:H cell:①Y; ②N; ③N; ④Y; ⑤Y
(Ito et al., 2022)	Human, mouse, C2C12 cell	M	COPD	Electrical pulse stimulation induces myotubular contraction to simulate movement	PINK1, Parkin	Insufficient Parkin-mediated mitochondrial autophagy leads to the accumulation of damaged mitochondria and excessive production of ROS, resulting in COPD-related sarcopenia.	A:Y; B:Y; C:Y; D:N; E:N	OCEBM:4, SYRCLE:M, cell: (1) Y; (2) Y; (3) Y; ④Y; ⑤N
(Yang et al., 2025)	Mouse, C2C12 cell, primary human skeletal muscle cell	M	Statin-induced myopathy	TOMM40, TOMM22 KD	PINK1, Parkin	The downregulation of TOMM40 and TOMM22 disrupts mitochondrial dynamics and increases mitochondrial autophagy, leading to skeletal muscle mitochondrial dysfunction.	A:N; B:N; C:Y; D:N; E:N	SYRCLE:H cell:①Y; ②N; ③Y; ④Y; ⑤N

## 4 Discussion

This scoping review, conducted in accordance with PRISMA-ScR guidelines and based on a systematic analysis of 113 original studies, progressively presents the current state of research on organelle-specific autophagy from basic mechanisms to physiological adaptations and pathological dysregulation. At the physiological level, organelle-specific autophagy primarily manifests as adaptive responses to stresses such as exercise, nutrition, and disuse. At the pathological level, organelle-specific autophagy often exhibits a biphasic transition from compensatory enhancement to decompensatory impairment, with disease and disease-stage specificity.

### 4.1 Research landscape and evidence distribution

Among the 113 studies included in this review, human studies accounted for only 15%, animal models 56%, and skeletal muscle cell lines 29%. By autophagy type, mitophagy dominated overwhelmingly with 98 studies (87%); reticulophagy and lysophagy each had five studies (4% each); pexophagy, ribophagy, and nucleophagy together accounted for less than 5%. This distribution clearly indicates that current skeletal muscle autophagy research is highly focused on mitochondria, with a serious underrepresentation of quality control mechanisms for other organelles. Regarding evidence level, among the 24 human studies included, 18 (75%) were cross-sectional observational studies or small case series (level 4), only three were RCTs (level 2b), and one was an individual RCT (level 1b); overall evidence was predominantly low-level observational, lacking high-quality interventional clinical trials. Among 88 animal studies, low RoB was found in 14 (16%), moderate RoB in 43 (49%), and high RoB in 30 (35%), indicating insufficient reporting of key bias controls such as randomization, allocation concealment, and blinding. For 46 cell experiments assessed by our five self-established criteria, the compliance rates were: 83% used TEM to confirm autophagosomes (gold standard), 28% used lysosomal inhibitors (bafilomycin A1, chloroquine, etc.) to validate autophagic flux, 72% used gene knockout/knockdown to verify mechanisms, 91% used skeletal muscle-derived cell lines (C2C12, L6, etc.), and 41% performed multi-time-point dynamic autophagy detection. This suggests that while current cell experiments are relatively standardized in autophagic morphology confirmation and cell model selection, they still show notable deficiencies in dynamic flux validation, indicating that future studies should strengthen monitoring of autophagic dynamic processes.

In summary, current conclusions on skeletal muscle organelle-specific autophagy are derived mainly from animal models and skeletal muscle cell experiments. Animal models allow tissue-specific genetic intervention, drug screening, and functional assessment under controlled conditions, providing essential experimental foundations for elucidating the causal relationships and dynamic patterns of autophagy in skeletal muscle physiological adaptation and pathological evolution. Skeletal muscle cell experiments enable precise manipulation of relevant pathways at the molecular level, facilitating the mechanistic dissection of autophagy initiation, recognition, and clearance steps. However, animal models cannot fully recapitulate the genetic background, metabolic characteristics, or complex regulatory networks during long-term disease progression in humans, while cell experiments lack the tissue microenvironment and cannot reflect the genuine effects of innervation, mechanical loading, cell–cell interactions, and systemic metabolic regulation on autophagy. Therefore, caution is warranted when extrapolating conclusions from existing animal and cell studies to the pathological mechanisms or intervention strategies for human skeletal muscle diseases.

### 4.2 Methodological limitations: from static markers to dynamic monitoring of autophagic flux

Among the 113 included studies, we assessed autophagic flux methodological indicators: 53% performed dual detection of LC3B and p62; 17% used lysosomal inhibitor blocking experiments to validate flux; 64% used TEM or tandem fluorescent probes (mRFP-GFP-LC3) to directly visualize autophagosome/autolysosome structures; 23% combined gene knockdown/overexpression or pharmacological agonists/inhibitors for bidirectional verification of autophagic function. These results indicate that while more than half of the studies focused on autophagosome formation, substrate accumulation, and static structural observation, less than one-quarter validated autophagic flux using lysosomal inhibitors or established causality through gene knockout/knockdown or pharmacological interventions. Consequently, it remains difficult to distinguish initiation, recognition, and clearance steps, limiting mechanistic depth. It is commendable that nine studies strictly adhered to a complete logical chain from static marker detection to dynamic flux validation, morphological confirmation, and causality verification: they not only used lysosomal inhibitors to distinguish autophagosome accumulation from genuine flux but also employed TEM or tandem probes to pinpoint the blocked step, and used gene knockout/knockdown or pharmacological interventions to reverse-validate the causal role of key molecules. These studies provide high-quality evidence for dissecting the dynamic regulation of skeletal muscle autophagy and set methodological benchmarks for the field.

### 4.3 Reversibility of interventions and distinction from accompanying phenomena

Among the 113 included studies, only 18% examined intervention reversibility, with most focusing on reambulation after immobilization. Although the number of relevant studies is limited, existing evidence clearly confirms that resuming weight-bearing or resistance training actively reverses the suppressed ribosome biogenesis and abnormally enhanced ribophagy induced by disuse, restoring total RNA content to baseline levels or even causing compensatory elevation (Figueiredo et al., 2021). Concurrently, reambulation reverses the overactivated mitophagy mediated by PINK1-Parkin, BNIP3/BNIP3L, etc., under disuse conditions, maintains mitochondrial fusion–fission balance, and gradually restores mitochondrial content and oxidative enzyme activity. This suggests that ribophagy and mitophagy, during the postimmobilization remobilization phase, actively achieve recovery and functional reconstruction of skeletal muscle atrophy by targeting and rectifying disuse-induced molecular disturbances and structural/functional decline. Given the current low proportion of reversibility studies and the relatively uniform intervention patterns, future efforts should expand reversibility validation protocols to further clarify the value of different interventions in reversing disuse-induced muscle injury.

### 4.4 Disease specificity and disease stage specificity of each organelle-specific autophagy

Under different pathophysiological states of skeletal muscle, each organelle-specific autophagy exhibits both disease-type specificity and disease-stage specificity in its initiation, recognition, and clearance steps, displaying multi-time-point temporal regulation characteristics. For instance, in skeletal muscle under exercise stress, acute eccentric exercise significantly upregulates the ER-phagy receptor FAM134B and autophagosome marker LC3-II in soleus muscle, peaking at 12 h and returning to baseline by 72 h, indicating rapid activation of ER-phagy recognition and formation (Jin et al., 2022); within 90 min after acute aerobic running, Atg7, Beclin1, Parkin, and BNIP3L are significantly elevated in tibialis anterior and extensor digitorum longus muscles, with enhanced autophagic flux (Vainshtein et al., 2015b); long-term endurance training significantly upregulates PINK1 and BNIP3 in vastus lateralis muscle, promoting mitochondrial recognition and clearance while reducing autophagosome accumulation, suggesting that steady-state mitochondrial quality control predominates in long-term exercise (Arribat et al., 2019). Overall, differences in autophagy initiation, recognition, and clearance steps under exercise stress result from the combined effects of exercise type, exercise duration, and target organelles.

Similarly, during early myogenic differentiation (myoblast fusion stage, approximately 4–7 days), FAM134B2 transcription is upregulated while FAM134B1 is downregulated, accompanied by increased LC3BII and decreased p62, suggesting that mitophagy initiation, recognition, and clearance steps are synchronously activated to support ER remodeling and myotube formation; as differentiation progresses to the maturation stage (after 10 days), ER structure stabilizes and FAM134B2 returns to basal levels (Buonomo et al., 2025). During early muscle development (7–14 days), BNIP3L is upregulated to initiate mitochondrial recognition; at mid-to-late stages (21–42 days), LC3BII continues to increase, p62 further decreases, and mitochondrial localization of BNIP3, PINK1, and Parkin significantly increases, indicating progressive strengthening of mitophagy initiation, recognition, and clearance steps (Rahman et al., 2025c). Overall, myogenic differentiation and development drive stage-specific organelle quality control by ER-phagy and mitophagy to accommodate ER structural stabilization and mitochondrial network remodeling, thereby maintaining myocyte homeostasis and structural maturation.

This is also reflected in skeletal muscle disuse. During short-term immobilization (24 h), mitophagy receptors such as BNIP3 and BNIP3L are significantly upregulated, indicating rapid activation of the recognition step; during mid-term immobilization (3–7 days), Parkin-mediated ubiquitin-dependent mitophagy is further enhanced, demonstrating sequential activation and synergy between receptor and ubiquitin pathways (Yamashita et al., 2021); concurrently, ribophagy is not activated immediately after immobilization but becomes significantly elevated only after 7 days of sustained disuse, suggesting that activation of the ribosome clearance step depends on a certain duration of disuse stimulus (Figueiredo et al., 2021). In the abnormal state following injury, the autophagic response displays stage transition characteristics: the early stage (14 days) is marked by increased Beclin-1, Atg4B, and LC3BII/I ratio and decreased p62, indicating enhanced autophagy initiation and autophagosome formation; the later stage (56 days) features sustained elevation of PINK1, Parkin, and VDAC1, indicating persistent activation of the mitophagy recognition step (Noh et al., 2024). Notably, muscle type further exacerbates heterogeneity in autophagic steps. In the gastrocnemius muscle, immobilization significantly increases BNIP3, BNIP3L, and FUNDC1, suggesting activation of Parkin-independent mitophagy recognition, along with elevated LC3BII and p62 protein levels, indicating increased autophagosome formation but impaired clearance; upon reambulation, all markers return to control levels, suggesting restoration of both recognition and clearance functions. In the tibialis anterior muscle, immobilization also elevates BNIP3L, LC3BII, and p62, again indicating blocked flux; upon reambulation, Parkin, BNIP3, BNIP3L, FUNDC1, LC3BII, and p62 all significantly increase, suggesting coactivation of both Parkin-dependent and Parkin-independent pathways and comprehensive enhancement of recognition, yet autophagic clearance function remains unrecovered (Deval et al., 2020). Overall, differences in initiation, recognition, and clearance steps of different organelle-specific autophagy during muscle disuse result from the combined effects of disuse duration, reambulation timing, and muscle type.

## 5 Conclusions

Following PRISMA-ScR guidelines, this scoping review systematically maps, via standardized tables, the current research landscape of mitophagy, reticulophagy, lysophagy, pexophagy, ribophagy, and nucleophagy in skeletal muscle physiological adaptation and pathological disturbance. To advance high-quality development and clinical translation in this field, future research should focus on the following: (1) strengthening molecular mechanism and functional studies of nonmitochondrial organelle-specific autophagy; (2) establishing standardized dynamic monitoring systems for autophagic flux; (3) conducting large-scale, prospective, interventional high-quality clinical studies; and (4) systematically analyzing sex differences and muscle fiber type specificity in autophagy regulation.
