# Supplementary material for: From homeostasis to pathology, organelle-specific autophagy in skeletal muscle: a PRISMA-ScR scoping review
Source: Front Physiol. 2026 May 7;17:1822139. doi: 10.3389/fphys.2026.1822139 (PMC13189897; doi:10.3389/fphys.2026.1822139)
Supplement: Supplementary file 1 [file DataSheet1.pdf]

1 Pubmed (1563)

((((((((((((Muscle*, Skeletal[MeSH Terms]) OR (Skeletal Muscle*[Title/Abstract])) OR (Muscle*, Voluntary[Title/Abstract])) OR (Voluntary Muscle*[Title/Abstract])) OR (Anterior Tibial Muscle[Title/Abstract])) OR (Muscle, Anterior Tibial[Title/Abstract])) OR (Tibial Muscle, Anterior[Title/Abstract])) OR (Gastrocnemius Muscle[Title/Abstract])) OR (Muscle, Gastrocnemius[Title/Abstract])) OR (Plantaris Muscle[Title/Abstract])) OR (Muscle, Plantaris[Title/Abstract])) OR (Soleus Muscle[Title/Abstract])) OR (Muscle, Soleus[Title/Abstract])) AND ((((((((((((Endoplasmic Reticulum Autophagy[MeSH Terms]) OR (Reticulophagy[Title/Abstract])) OR (ER-Phagy[Title/Abstract])) OR (ER Phagy[Title/Abstract])) OR (autophagy of the endoplasmic reticulum[Title/Abstract])) OR (autophagy of the ER[Title/Abstract])) OR (ER autophagy[Title/Abstract])) OR (((((Mitophagy[MeSH Terms]) OR (Mitochondrial Degradation[Title/Abstract])) OR (autophagy of mitochondrion[Title/Abstract])) OR (mitochondrion autophagy[Title/Abstract])) OR (mitochondrion degradation[Title/Abstract]))) OR ((((((Pexophagy[MeSH Terms]) OR (Peroxisome Autophagy[Title/Abstract])) OR (Peroxisomal Autophagy[Title/Abstract])) OR (autophagy of peroxisome[Title/Abstract])) OR (peroxisome degradation[Title/Abstract])) OR (peroxisome disassembly[Title/Abstract]))) OR ((((Lysophagy[MeSH Terms]) OR (Lysosome Autophagy[Title/Abstract])) OR (Lysosomal Autophagy[Title/Abstract])) OR (Autophagy of Lysosome[Title/Abstract]))) OR (((Nucleophagy[MeSH Terms]) OR (Nuclear Autophagy[Title/Abstract])) OR (Autophagy of the Nucleu[Title/Abstract]))) OR ((((Ribophagy[MeSH Terms]) OR (Ribosome Autophagy[Title/Abstract])) OR (Ribosomal Autophagy[Title/Abstract])) OR (Autophagy of Ribosome[Title/Abstract])))

2 EMBASE(1086)

#1

'Muscle*, Skeletal' OR 'Skeletal Muscle*' OR 'Muscle*, Voluntary' OR 'Voluntary Muscle*' OR 'Anterior Tibial Muscle' OR 'Muscle, Anterior Tibial' OR 'Tibial Muscle, Anterior' OR 'Gastrocnemius Muscle' OR 'Muscle, Gastrocnemius' OR 'Plantaris Muscle' OR 'Muscle, Plantaris' OR 'Soleus Muscle' OR 'Muscle, Soleus'

#2

'Endoplasmic Reticulum Autophagy' OR 'Reticulophagy' OR 'ER-Phagy' OR 'ER Phagy' OR 'autophagy of the endoplasmic reticulum' OR 'autophagy of the ER' OR 'ER autophagy'

#3

'Mitophagy' OR 'Mitochondrial Degradation' OR 'autophagy of mitochondrion' OR 'mitochondrion autophagy' OR 'mitochondrion degradation'

#4

'Pexophagy' OR 'Peroxisome Autophagy' OR 'Peroxisomal Autophagy' OR 'autophagy of peroxisome' OR 'peroxisome degradation' OR 'peroxisome disassembly'

#5

'Lysophagy' OR 'Lysosome Autophagy' OR 'Lysosomal Autophagy' OR 'Autophagy of Lysosome'

#6

'Nucleophagy' OR 'Nuclear Autophagy' OR 'Autophagy of the Nucleu'

#7

‘Ribophagy’ OR ‘Ribosome Autophagy’ OR ‘Ribosomal Autophagy’ OR ‘Autophagy of Ribosome’
#8=#2 OR #3 OR #4 OR #5 OR #6 OR #7
#9=#1 AND #8

3 Cochrane Library (57)
#1
Muscle*, Skeletal or Skeletal Muscle* or Muscle*, Voluntary or Voluntary Muscle* or Anterior Tibial Muscle or Muscle, Anterior Tibial or Tibial Muscle, Anterior or Gastrocnemius Muscle or Muscle, Gastrocnemius or Plantaris Muscle or Muscle, Plantaris or Soleus Muscle or Muscle, Soleus
#2
Endoplasmic Reticulum Autophagy or Reticulophagy or ER-Phagy or ER Phagy or autophagy of the endoplasmic reticulum or autophagy of the ER or ER autophagy
#3
Mitophagy or Mitochondrial Degradation or autophagy of mitochondrion or mitochondrion autophagy or mitochondrion degradation
#4
Pexophagy or Peroxisome Autophagy or Peroxisomal Autophagy or autophagy of peroxisome peroxisome degradation or peroxisome disassembly
#5
Lysophagy or Lysosome Autophagy or Lysosomal Autophagy or Autophagy of Lysosome
#6
Nucleophagy or Nuclear Autophagy or Autophagy of the Nucleu
#7
Ribophagy or Ribosome Autophagy or Ribosomal Autophagy or Autophagy of Ribosome
#8=#2 or #3 or #4 or #5 or #6 or #7
#9=#1 and #8

4 Web of Science (1114)
#1
TS=(“Muscle*, Skeletal“or“Skeletal Muscle*“or“Muscle*, Voluntary“or“Voluntary Muscle*“or“Anterior Tibial Muscle“or“Muscle, Anterior Tibial“or“Tibial Muscle, Anterior“or“Gastrocnemius Muscle“or“Muscle, Gastrocnemius“or“Plantaris Muscle“or“Muscle, Plantaris“or“Soleus Muscle“or“Muscle, Soleus“)
#2
TS=(“Endoplasmic Reticulum Autophagy“or“Reticulophagy“or“ER-Phagy“or“ER Phagy“or“autophagy of the endoplasmic reticulum“or“autophagy of the ER“or“ER autophagy“)
#3
TS=(“Mitophagy“or“Mitochondrial Degradation“or“autophagy of mitochondrion“or“mitochondrion autophagy“or“mitochondrion degradation“)
#4
TS=(“Pexophagy“or“Peroxisome Autophagy“or“Peroxisomal Autophagy“or“autophagy of peroxisome“or“peroxisome degradation“or“peroxisome disassembly“)

#5

TS=(“Lysophagy “or “Lysosome Autophagy “or “Lysosomal Autophagy “or “Autophagy of Lysosome “)

#6

TS=(“Nucleophagy “or “Nuclear Autophagy “or “Autophagy of the Nucleu “)

#7

TS=(“Ribophagy “or “Ribosome Autophagy “or “Ribosomal Autophagy “or “Autophagy of Ribosome “)

#8=#2 or #3 or #4 or #5 or #6 or #7

#9=#1 AND #8
